# Historic Late Blight Outbreaks Caused by a Widespread Dominant Lineage of *Phytophthora infestans* (Mont.) de Bary

**DOI:** 10.1371/journal.pone.0168381

**Published:** 2016-12-28

**Authors:** Amanda C. Saville, Michael D. Martin, Jean B. Ristaino

**Affiliations:** 1 Department of Entomology and Plant Pathology, North Carolina State University, Raleigh, North Carolina, United States of America; 2 Department of Natural History, NTNU University Museum, Norwegian University of Science and Technology (NTNU), Trondheim, Norway; 3 Formerly Centre for GeoGenetics, Natural History Museum of Denmark, University of Copenhagen, Copenhagen, Denmark; Agriculture and Agri-Food Canada, CANADA

## Abstract

*Phytophthora infestans* (Mont.) de Bary, the causal agent of potato late blight, was responsible for the Irish potato famine of the 1840s. Initial disease outbreaks occurred in the US in 1843, two years prior to European outbreaks. We examined the evolutionary relationships and source of the 19^th^-century outbreaks using herbarium specimens of *P*. *infestans* from historic (1846–1970) and more recent isolates (1992–2014) of the pathogen. The same unique SSR multilocus genotype, named here as FAM-1, caused widespread outbreaks in both US and Europe. The FAM-1 lineage shared allelic diversity and grouped with the oldest specimens collected in Colombia and Central America. The FAM-1 lineage of *P*. *infestans* formed a genetic group that was distinct from more recent aggressive lineages found in the US. The US-1 lineage formed a second, mid-20^th^ century group. Recent modern US lineages and the oldest Mexican lineages formed a genetic group with recent Mexican lineages, suggesting a Mexican origin of recent US lineages. A survey of mitochondrial haplotypes in a larger set of global herbarium specimens documented the more frequent occurrence of the HERB-1 (type Ia) mitochondrial haplotype in archival collections from 1866–75 and 1906–1915 and the rise of the Ib mitochondrial lineage (US-1) between 1946–1955. The FAM-1 SSR lineage survived for almost 100 years in the US, was geographically widespread, and was displaced first in the mid-20^th^ century by the US-1 lineage and then by distinct new aggressive lineages that migrated from Mexico.

## Introduction

Movement of plant pathogens into new geographic ranges and expansion into new hosts is a major factor in the emergence of novel virulent lineages that threaten food security [1]. The oomycete pathogen *Phytophthora infestans* (Mont.) de Bary exemplifies this threat and causes potato late blight. This disease is the most important biotic constraint to potato production globally [2]. Historically, potato late blight caused massive food insecurity during Ireland’s famine of the 1840s [3]. Long distance movement of the pathogen into new geographic areas occurs primarily via the movement of infected plant materials including potato tubers, tomato fruit, and transplants.

The current global population structure of *P*. *infestans* results from a series of migrations and displacements of clonal lineages [4–9]. While populations in Mexico, the Netherlands, Scandinavia, and other parts of Europe are sexual [6, 10–12], the genetic structure of *P*. *infestans* in the US and Canada has been simpler and largely consists of new introductions of a few multilocus genotypes that have been displaced over time [13–15]. This is despite the occurrence of both A1 and A2 mating types in some areas of the US and Canada and ephemeral sexual populations [16, 17].

Although we have improved our ability to track recent outbreaks of late blight [6, 14], an unresolved question of significance is the source of inoculum for historic 19^th^-century US and European outbreaks of the disease. Some data support a Mexican source of the disease [7, 18, 19] while other data support an Andean source of the disease [3, 20–22] or a US metapopulation emergence of the pathogen [23]. In the US, the disease first appeared in 1843, two years prior to European outbreaks, around the ports of Philadelphia and New York [3, 24]. Early scientists believed the disease originated in the Andean region of South America and cite reports from New World explorers indicating that the disease was known to native people in the Andes [22, 25, 26]. The burgeoning bat guano trade for fertilizer from Peru and failure of the European potato crop due to dry rot resulted in increased shipments of potatoes from the Andean region into both US and European ports [3]. In addition, the closely related hybrid species *P*. *andina* occurs only in the Andean region and shares an ancestral haplotype lineage with famine-era lineages of *P*. *infestans* [27–30], thus suggesting an Andean outbreak source.

A single clonal lineage named US-1 (Ib mitochondrial DNA (mtDNA), which was globally distributed in the mid-20^th^ century, was suspected to be the cause of ‘famine-era’ outbreaks of *P*. *infestans* [7] until PCR amplification of mitochondrial genes from 19^th^-century samples documented an entirely different Ia mtDNA haplotype [8,9]. We concluded that another migration must have led to the widespread occurrence of the US-1 clonal lineage in the mid-20^th^ century [31]. Two independent labs recently sequenced whole genomes from historic New and Old World *P*. *infestans* collected between 1845 and 1896 from herbarium samples [23, 32, 33, 34]. Examination of these genomes indicated the presence of a unique genotype, which was named HERB-1 for both mitochondrial and nuclear genomes by Yoshida et al. after examination of one North American and ten European genomes. [23]. Although it was suggested that the HERB-1 lineage was extinct or rare in modern populations [23, 35], Martin et al. [33] sequenced 44 additional mitogenomes and determined the HERB-1/Ia mitochondrial lineage to be extant in both Mexican and South American populations. Martin et al. found that the HERB-1 mitochondrial lineage was also present in the Andean hybrid species *P*. *andina* Adler & Flier, sp. nov in a study that included genomic data from both Yoshida et al. and Martin et al. and that the mitochondrial lineage diverged into several clades [23, 27, 32, 33]. Thus, HERB-1 is not exclusive to *P*. *infestans*.

We collected a larger set of historic specimens of *P*. *infestans* from North, Central, and South America (S1 Table) than previous studies and examined the population genetic structure to confirm that New and Old World outbreaks were caused by the same lineage of *P*. *infestans* and to determine how widespread the lineage was. We sequenced nuclear and mitochondrial genes as well as 12 nuclear single sequence repeat (SSR) loci to resolve the population structure and evolutionary relationships of historic New and Old World *P*. *infestans* and to characterize the SSR lineage from original outbreaks The primary objectives of our work were to: 1) Examine the evolutionary relationships of *P*. *infestans* from the first historic US late blight outbreaks; 2) Compare the genetic structure of these populations to historic European *P*. *infestans*; 3) Examine the genetic relationships of historic to modern US aggressive lineages; and 4) Explore migration scenarios that best describe the source of the first US historic outbreaks of *P*. *infestans*.

## Materials and Methods

### Sampling

*Phytophthora infestans* was sampled from both historic US and European herbarium vouchers as well as modern isolates obtained from cultures derived from infected host tissue. A total of 183 samples were analyzed from historic US (1855–1958), European (1846–1970), South American (1913–1929), Central American (1941–1956), Mexican (1948–1966), and more recent global sources (1992–2014) (S1 Table). The historic collections tested (*n* = 66) include the oldest existing US specimen, collected in 1855, and one of the oldest existing European specimens, collected in 1846. Modern samples collected in North America (*n* = 34) included samples from the early 1950s-1990s (US-1, US-6, US-7) and recent US lineages (US-8, US-11, US-22, US-23, US-24)[6, 14, 15]. Sampled regions outside of North America (*n* = 83) include South America (Bolivia, Brazil, Peru, Ecuador, Colombia), Central America (Costa Rica, Guatemala, Nicaragua), Ireland, Canada, and Mexico. Two historic cultures (P445 and PA 222) of *P*. *infestans* collected in the 1950s and 1960s were shared by Mannon Gallegly. The P445 isolate is an A2 mating type and was collected in Mexico and is notable for its use in the US Cold War late blight research at Fort Detrick. The PA 222 is an A1 mating type and was collected in Pennsylvania.

### Data analysis

For specific analyses, samples were divided into eight populations: historic US (USHist), historic European (EUHist), US-1 lineages (US-1; Ib mtDNA haplotype), modern US lineages (USAGG), South America (SA), Central America (CA), Mexico (MEX), and Ireland (IRE). With the exception of US-1, all groups were divided based on spatio-temporal boundaries.

### DNA extraction, PCR and sequencing

DNA from modern isolates was extracted using either a hexadecyltrimethylammonium bromide (CTAB) method [8] or a modification of the quick sodium hydroxide extraction method described by Wang et al. [36]. DNA from herbarium samples was extracted using a modified CTAB method and a Qiagen DNEasy Plant Mini Kit (Qiagen, Valencia, CA) [8]. All work with historic DNA was done in BSL-2 laboratories in the Phytotron and the Genomic Sciences Laboratory at NC State with separate reagents and equipment. No modern DNA was used in these labs. A subset of the samples used in this study were subjected to next-generation sequencing analyzed at the Centre for GeoGenetics (University of Copenhagen). The subsequent genomic analyses have been published [32,33].

Two nuclear loci (*ras* and *PiAVR2*) and one mitochondrial (P3) locus were sequenced (S2 Table). For the nuclear locus *ras*, two regions were amplified including intron 1 (*Intron Ras*; 349 bp with IRF/IRR) located in the 5’ untranslated region of the gene and a 600 bp portion (with RASF/RASR) covering part of exon 3, exon 4, exon 5, part of exon 6, and introns 3 and 4 [21, 37]. For herbarium samples, 224 bp of intron 1 were sequenced with IRF/IRR and the larger 600 bp region was sequenced with two sets of primers (RAS1F/RAS1R and RAS2F/RAS2R) that amplified the polymorphic sites in two smaller regions (162 and 245 bp) (S2 Table). The AVR gene, *PiAVR2*, was amplified with primers AVR2F1 and AVR2R2 [38]. For herbarium samples, a smaller region (200 bp) nested within the gene was amplified with primers AVR2F4 and qRT-PCR-R. For the mitochondrial locus P3 for modern isolates, a 1446 bp region was amplified with primers F3/R3 [39]. The P3 region includes the genes *rp114*, *rp15*, and *tRNAs*. For herbarium samples, a smaller 492 bp region nested within the P3 region was amplified with primers P3H4F/ P3H6R [8, 9].

Martin et al. [27] documented the presence of a single SNP within the mitochondrial genome that distinguished the HERB-1 mitochondrial lineage from all other known lineages. Primers were developed (nad11F/nad11R, S2 Table) that amplified a 180 bp region around the target SNP. Amplicons produced were sequenced and utilized to detect the presence of the HERB-1 mitochondrial lineage in historic samples. The complete mitogenome sequence of HERB-1 has been submitted to the Sequence Read Archive [27]. The mitochondrial haplotype of *P*. *infestans* in a larger set of herbarium samples was identified using methods reported elsewhere [8, 39]. The frequency of occurrence of the Ib and Ia/HERB-1 mitochondrial lineages was calculated over time.

PCR reactions were performed in 50 μL volumes for each sample. Each reaction contained 5 μL of 10X PCR buffer (Genesee, San Diego, CA), 2.5 μL dNTPs (2 mM per nucleotide), 2 μL each 10 μM forward and reverse primer, 1.8 μL MgCl_2_ (50 mg/mL), 0.25 μL BSA (20 mg/mL), 0.2 μL Taq (5 U/μL)(Genesee, San Diego, CA), and 5–10 ng of genomic DNA. For herbarium samples, reaction volumes were decreased to 25 μL. Thermal cycling conditions for nuclear genes were 96°C (1 min); then 35 cycles of 96° (1 min), 55° (1 min), 72° (2 min); and a final extension of 72° (10 min). For mitochondrial gene regions, thermal cycling conditions followed Griffith and Shaw [39]. PCR reactions were run at least twice.

Gene sequences were determined for some of the target loci (S2 Table) within Illumina-sequenced *P*. *infestans* isolates by creating multiple sequence alignments of genotype calls from Martin et al. [27]. BAM files of sequences mapped to the T30-4 reference genome (available on the Sequence Read Archive under accession SRP055472). Read alignments to the reference genome had previously been optimized with the RealignerTargetCreator and IndelRealigner tools included in the software Genome Analysis Toolkit (GATK) v1.3 [40]. GATK was used to perform the genotype calling, requiring a minimum PHRED-scaled genotype quality score of 20.0. Genotypes not fulfilling this requirement were masked from the alignment.

### SSR genotyping

*P*. *infestans* SSR loci were genotyped using a modified version of the protocol for 12-plex single sequence repeat genotyping as described previously [41]. The Qiagen Type-It Microsatellite PCR kit (Qiagen Corporation, Valenica CA) was used for PCR reactions, and sample volumes were modified to run a 12.5 μL reaction by using 6.25 μL 2X Type-It Master Mix, 1.25 μL of a 10X multiplex primer master mix, 4 μL PCR grade water, and 1–2 μL of template DNA (5–10 ng). Thermal cycling conditions followed Danies et al. [16]. Fragments were analyzed on an Applied Biosystems 3730xl DNA analyzer at the Genomic Sciences Laboratory at North Carolina State University. Alleles were scored manually using Peak Scanner 2 (Applied Biosystems, Foster City, CA), and fragment lengths were rounded to the nearest whole number for analysis.

### SSR genotyping analyses

Analysis of SSR genotypes was conducted using the program Structure v.2.3.3 [42]. The data were run using a 20,000 repeat burn-in and 1,000,000 MCMC repeats under an admixture model. Independent runs of the model used *K* values from 1 to 10 with 20 replicate runs at each value of *K*. The optimal *K* was estimated using the Evanno method in the web tool Structure Harvester [43]. In addition, the optimal K was inferred through direct observation of groupings of the samples by their assigned Q values. All runs for the optimal K values, as well as non-optimal K values, were averaged using CLUMPP v. 1.1.2 [44] and visualized with the program Distruct v. 1.1. [45]. The geographic distribution of SSR genotypes based on Structure results was examined by mapping samples based on *K* value onto maps of Europe, Latin America, and the United States. To further visualize groupings, a discriminant analysis of principal components (DAPC) was conducted using the R library adegenet [46]. The R library Poppr [47] was used to infer population statistics on a clone corrected dataset of the SSR genotypes.

### Gene sequence analysis

All statistical analyses of the nucleotide sequences were performed in SNAP Workbench version 2.0 [48]. All sequences were aligned manually and edited using BioEdit [49]. Multiple sequence alignment was also performed in Clustal W [50]. Polymorphisms were examined on the chromatograms and heterozygous sites were determined. Sequences were collapsed into unique haplotypes using SNAP Map [51] after removing insertions and deletions (indels) from each of the aligned multilocus data sets and excluding infinite-sites violations. Resultant haplotype data sets were used to examine the overall support or conflict among the variable sites in the DNA sequence alignment. A site compatibility matrix was generated from each haplotype data set using SNAP Clade [52]. Compatibility matrices were used to examine compatibility/incompatibility among all variable sites, with any resultant incompatible sites removed from the data set. Data sets were also evaluated using Kwarg [53] for estimating the minimum number of recombination events and constructing ancestral recombination graphs (ARG). Conflicting data partitions or putative recombinant haplotypes were excluded from further analyses, except when testing for population subdivision using Hudson’s test statistics as recombination increases the power of these tests [54–56]. Non-recombining data sets were collapsed into unique haplotypes excluding infinite-sites violations using SNAP Map. The *ras* dataset contained 12 of the 14 documented SNPs in the dataset, while no recombination was detected in *PiAVR2* or in P3.

### Neutrality tests and population subdivision

The nuclear and mitochondrial DNA sequences were analyzed using Arlequin [57]. For each locus in each population, the population mean mutation rate per nucleotide site θ_w_ was calculated using Watterson’s θ [58], based on the number of segregating sites, *s*, and the average pairwise nucleotide diversity, π [59] were estimated. Different tests of neutrality including Tajima’s D and Fu’s Fs statistic [60, 61] were performed in order to determine if the data were consistent with the expectations of the neutral model of molecular evolution.

### Coalescent analysis

Ancestral recombination graphs and coalescent analyses were generated using the loci *ras*, *PiAVR2*, and P3. Coalescent analyses were conducted using genetree [62] as implemented in SNAP Workbench. Watterson’s theta (θ) was used for estimations of the population neutral mutation rate, and was estimated for each dataset using the program simple theta [63]. Analyses were performed as subdividing populations with 10 million runs. For the purposes of subdivision, samples were designated as South American (SA) or not South American (NSA). The trees were generated five (P3) and fifteen (*ras*) times, each using different random seeds to evaluate convergence. The representative tree was chosen by examining the consensus of topology and mutation structure between all trees.

### Gene flow and migration

We used the *ras* sequence data and IM to calculate migration rates between populations for US historic, South American, and Mexican lineages and US-1, South American, and Mexican lineages [63]. These results were corroborated using SSR genotypes with tests of potential migration patterns using Approximate Bayesian Comparison (ABC), as implemented in the program DIYABC v. 2.0.4 [64]. Tested migration scenarios for both US historic and US-1 populations included direct divergence from South America or Mexico, admixture with South America and Mexico populations, and admixture between South America or Mexico and an unsampled population. To further explore US and EU historic population migration scenarios, an additional migration scenario set was tested using US historic, EU historic, and South American populations. Parameter range priors were initialized with values from Goss et al. [65] and then iteratively modified to better fit our data (S3 Table). Scenario probabilities were determined through comparison of the observed dataset to simulated datasets generated by DIYABC. A logistic regression of these differences was computed using ten proportions of the simulated dataset as the dependent variable and corresponding differences between the observed and simulated datasets as the independent variable. The highest value was taken as the scenario’s overall probability. Confidence in the four highest scenarios was evaluated using type I and type II error tests, in which the data were compared against 500 simulated data sets and the number of times the scenario in question was correctly or incorrectly applied to the data was determined.

## Results

### Population structure

Samples were divided into eight populations by geography and time: historic US (USHist), historic European (EUHist), US-1 lineages (US-1; Ib mtDNA haplotype), modern US lineages (USAGG), South America (SA), Central America (CA), Mexico (MEX), and Ireland (IRE). A total of 179 multilocus genotypes (MLG) were detected within 12 microsatellite loci. The greatest number of MLGs was observed within the US historic (USHist), US Aggressive (USAGG) and South America (SA) populations, while the least number of MLGs was observed among modern Irish (IRE) populations. USHist, USAGG and SA lineages had the highest MLG diversity indices. The Index of Association (I_a_) was calculated for clone corrected data (S4 Table). Populations from Mexico (MEX) had the lowest I_a_, indicating the least inter-locus linkage, and the hypothesis of sexual reproduction could not be rejected. The highest I_a_ was observed in Central America (CA) and SA populations, indicating no linkage among markers and clonal populations.

**Fig 1:**
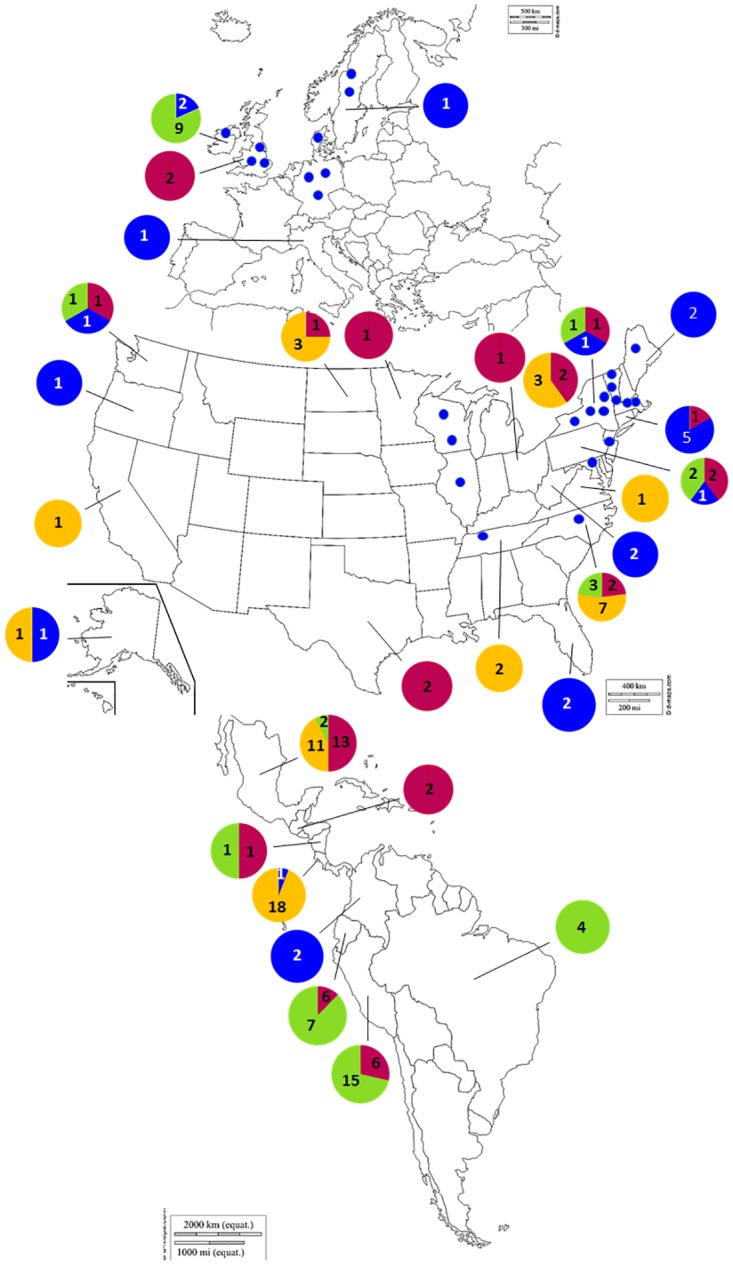
Geographic distribution of multilocus SSR genotypes of *Phytophthora infestans*. Small blue circles indicate FAM-1 SSR lineage found before 1900, and larger circles indicate samples collected after 1900. Samples were assigned to genetic clusters based on the highest Q value and correspond to *K* = 4 groups by color as in Fig 2. The FAM-1 lineage was geographically widespread. Samples are located by state in the US and by country in the rest of the world. Maps reprinted from www.d-maps.com under a CC BY license, with permission from Daniel Dalet, original copyright 2007–2016.

**Fig 2:**
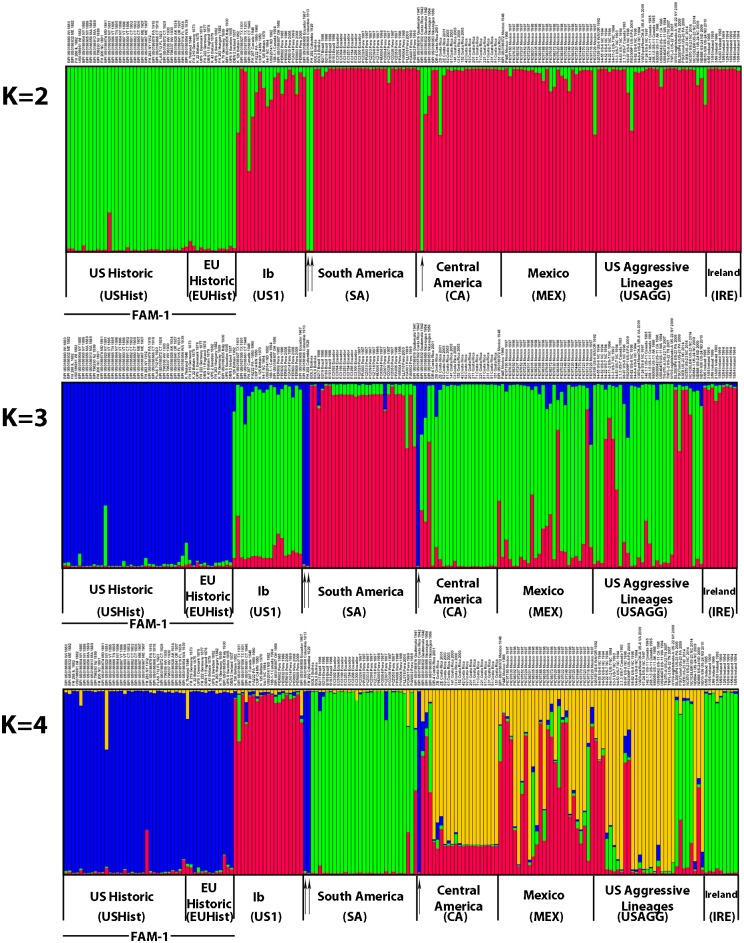
Structure analysis of 12 plex SSR loci from *Phytophthora infestans*. Optimal value for *K* was inferred to be *K* = 2 based on analysis by Structure Harvester and *K* = 4 based on evaluation of probabilities [43]. The oldest SSR lineage, FAM-1 consisted of samples from US (1855–1958) and EU Historic (1846–1970) outbreaks. Black arrows indicate specimens from the oldest South and Central American outbreaks that were also identified as FAM-1 lineages. A complete list of samples is shown in the S1 Table.

**Fig 3:**
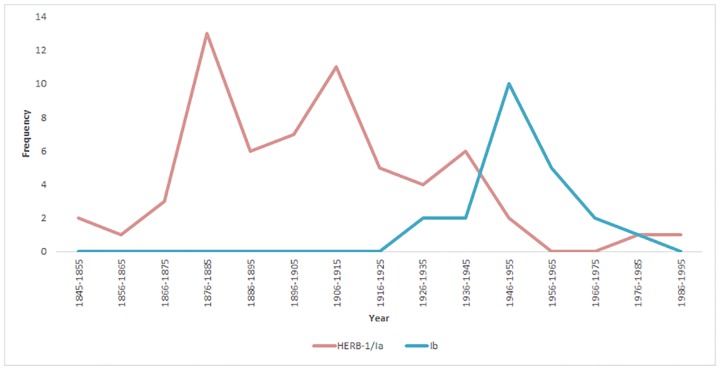
Number of HERB-1/Ia and Ib mitochondrial haplotypes (1845 to 1995) of *Phytophthora infestans* in herbaria collections. Mitochondrial haplotypes were identified from *P*. *infestans* in herbarium collections shown in the S1 Table. Mitochondrial lineages were determined by sequencing the P2 mitochondrial region around the *Msp*1 restriction site present in type Ib haplotypes and the rare allele found in *Nad11* in the HERB-1 mitogenome [27, 39].

From these genotypes, we inferred population structure using Structure and used both Structure Harvester and direct observation of cluster assignment probabilities for grouping individuals. The optimal *K* value determined by Structure Harvester was *K* = 2, and via observation of probabilities was *K* = 4. Both the New World historic (USHist) and Old World historic (EUHist) outbreaks belonged exclusively to the same genetic cluster, representing a nuDNA SSR lineage that was named FAM-1 (Fig 1). This FAM-1 SSR lineage consisted of the oldest specimens and was well differentiated from the remaining three clusters at all values of *K* (Fig 2). In addition to all USHist and EUHist samples, this genetic group also contained *P*. *infestans* genotypes recovered from the two oldest South American (SA) samples from Colombia collected in 1913 and 1929 and the oldest sample from Costa Rica collected in 1942. With *K* = 4, the US-1 genotype (mtDNA haplotype Ib) formed a second distinct genetic group (Figs 1 and 2). The US-1 genotype was found among global populations from the US, Europe, and South America from 1931–1994. US-1 was observed in the greatest numbers in the herbarium records from the post-WWII era from 1946–1955 (Fig 3). The historic isolate PA222, collected in the US and studied during the US Cold War-era bioweapons program in the 1950s, was also assigned to the US-1 cluster. At both *K* = 3 and *K* = 4, a third group was observed that included the US-23 genotype and genotypes from South America and Ireland (Fig 2). A fourth genetic group was comprised of several historic and recent genotypes from Mexico (MEX), Central America (CA), and the rest of the US Aggressive (USAGG) genotypes (S1 Table).

**Fig 4:**
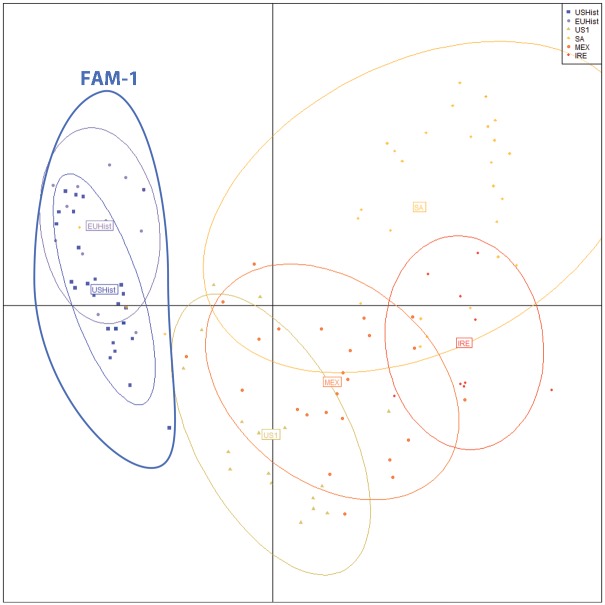
Discriminant Analysis of Principal Components (DAPC) plot of 12-plex SSR loci from modern and historic samples of *Phytophthora infestans*. Groups are represented by color/symbol as well as by inertia ellipses. Each point represents the SSR genotype of a single sample. Individuals are grouped by US Historic (USHist 1855–1958); European Historic (EUHist 1846–1970); US1: samples identified as mtDNA haplotype Ib or clonal lineage US-1; South American (SA): samples collected in South America; Mexico (MEX): samples collected in Mexico; and Ireland (IRE): samples collected in Ireland. The FAM-1 SSR lineage consisted of US and EU historic samples.

Discriminant analysis of principal components (DAPC) was conducted utilizing SSR data. USHist and EUHist populations formed two overlapping populations largely separate from all other groups. The FAM-1 lineage shared allelic diversity with some samples from SA populations (Fig 4). The largest inertia ellipse contained samples from SA and smaller subsets of samples from MEX, US-1 and IRE clustered with SA populations. The US-1 lineage contained samples from both MEX and SA (Fig 4).

### Nuclear and mitochondrial sequence variability

A total of 1259 nucleotides were sequenced, consisting of: 680 nucleotides of the nuclear *ras* gene (intron 1 and exon regions 3–6); 200 nucleotides of the *PiAVR2* gene; and 379 nucleotides in the mitochondrial genome region P3 (*rpl*14, *rpl*5 and tRNAs) (S5 Table). Twelve segregating nucleotide sites were identified in the *ras* gene, including 5 in intron 1 and another 5 in the exons 3–6 that were phylogenetically informative (S6 Table). A total of eleven haplotypes were observed in *ras* gene sequences, including one additional segregating site not observed previously [21](S5 and S6 Tables). The greatest number of haplotypes (eight) was found in the IRE populations. Only two haplotypes was found among EUHist, US-1, and CA populations (S5 Table).

One synonymous substitution site and 4 nonsynonymous substitutions were found in exons 3–6 of the *ras* gene (S6 Table). Populations from IRE, USAGG, and SA had higher nucleotide diversity (π) and mean mutation rates (θ_W_) than populations from MEX (S5 Table). All populations were determined to be neutral based on Tajima’s D and Fu’s F_s_ statistics. However, the presence of subdivision was detected through the use of Hudson’s statistics, suggesting the populations do not conform to Hardy-Weinberg equilibrium, which is expected at least within clonal lineages. Gene flow between the USHist and EUHist populations was evident (Ks = .0593, Kst = .005, p>0.05) (S7 Table).

One segregating nucleotide site was identified in *PiAVR2*, and it was phylogenetically informative and resulted in a nonsynonymous substitution site and two haplotypes were observed (S4 Table). Populations from MEX, EUHist, and CA had higher nucleotide diversity and mean mutation rates (θ_W_) than populations from USHist, US-1, SA and IRE (S5 Table).

There were 4 segregating sites identified in the P3 region (S5 Table). Four haplotypes were found in the mitochondrial P3 sequences and all were previously observed by Gómez-Alpizar et al. [21]. Populations from IRE, USAGG and SA had higher nucleotide diversity and mean mutation rates (θ_W_) compared to those from MEX (S5 Table). Since there was one dominant haplotype found in USHist, EUHist, US-1 and CA populations, estimates of π and θ_W_ values were null for these loci. For the populations for which neutrality tests could be conducted, all were determined to be neutral.

### Phylogeographic source of historic lineages

**Fig 5:**
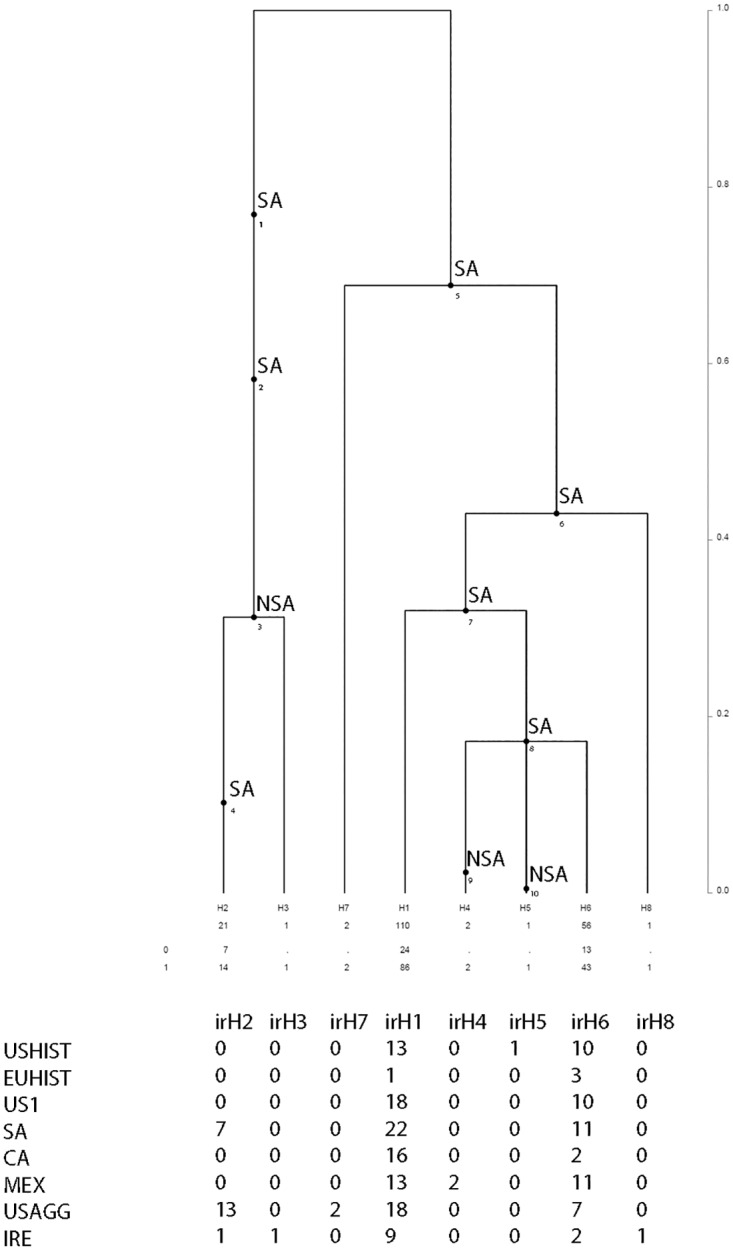
Coalescent analysis using the *ras* gene from modern and historic samples of *Phytophthora infestans*. The distribution of mutations for South American (SA) and non-South American (NSA) populations are shown. The frequency of occurrence of haplotypes and the times to the most recent common ancestor are shown in coalescent units for each population examined including US Historic (USHist), European Historic (EUHist), US-1, South American (SA), Central American (CA), Mexican (MEX), US Aggressive (USAGG) and Ireland (IRE). Times are not to scale. Estimates were based on 10 million coalescent simulations and fifteen independent runs to ensure convergence. Simulations were performed assuming constant population size and utilized the largest nonrecombining block of data.

The largest nonrecombining block of sequence data from the *ras* gene was utilized for coalescent analysis, and 8 haplotypes were identified (Fig 5). One of the 8 haplotypes (irH5) was unique to historic populations, and was observed only within the USHist population. We used the coalescence process to infer the distribution of mutations on the branches of the tree [21]. The most ancestral mutations observed within USHist and EUHist populations were assigned a South American origin (Fig 5). irH2 and irH3, present in the IRE, USAGG, and SA clusters, formed one of two major lineages identified and the most ancestral mutations from these haplotypes were of South American (SA) origin (Fig 5).

Analyses of ancestral recombination indicated that the one haplotype unique in historic populations was not generated through recombination (S1 Fig). This haplotype (H6), present in USHist, diverged prior to the most ancestral recombination event (S1 Fig). Evidence for a more recent recombination event that gave rise to haplotype H4, unique to USAGG populations, was also observed (S1 Fig). This haplotype was found in the US-11 lineage, a putative recombinant lineage that is found in tomato in the US [6].

Two haplotypes were observed within the *PiAVR2* locus, and they were shared between all historic and modern populations except IRE, in which only H1 was observed (S2 Fig and S8 Table). Four haplotypes were observed within the P3 mitochondrial gene region (*rpl14*, *rp15*, and *tRNAs*), including one haplotype (H4) unique to the SA population and one (H3) unique to the MEX population (S3 Fig and S8 Table). The ancestral recombination graphs indicated no recombination among *PiAVR2* or the mitochondrial loci (S3 Fig).

### Migration pathways from SA

Mean population mutation rates and numbers of migrants into populations were determined for USHist, EUHist, MEX, SA, and US-1 populations by comparing migrations between paired populations using the *ras* locus, which was the most phylogenetically informative locus (S9 Table). A greater mean number of migrants was observed moving into EUHist than into USHist for both *ras* (m_2_ = 6.63 vs m_1_ = 4.59) and *PiAVR2* (m_2_ = 6.99 vs m_1_ = 5.05) (S9 Table). Mean population mutation rates were also higher for EUHist than USHist populations. Thus, asymmetric migration into EUHist populations was observed.

Although migration into both USHist and MEX populations was detected, a higher mean population mutation rate and higher mean number of migrants was observed into USHist (m_1_ = 6.34) than MEX (m_2_ = 5.70) populations for *ras*, indicating asymmetric migration (S9 Table). Additionally, a higher mean population mutation rate and mean number of immigrants was observed into SA (m_2_ = 6.25) than USHist (m_1_ = 4.62), also indicating asymmetric migration. Only a slightly higher mean number of migrants and symmetrical migration between SA and USHist populations was observed for *PiAVR2* (m_2_ = 6.49, m_1_ = 5.87).

Both MEX and SA contributed migrants into US-1. Higher mean numbers of migrants into MEX than US-1 populations (m_2_ = 7.02 vs m_1_ = 5.03) and SA than US-1 populations (m_2_ = 6.18 vs m_1_ = 4.85) was observed for *ras*. Similarly, a higher mean number of migrants into MEX than US-1 populations (m_2_ = 6.48 vs m_1_ = 4.45) and SA than US-1 populations (m_2_ = 6.54 vs m_1_ = 5.78) was observed for *PiAVR2* (S9 Table).

**Fig 6:**
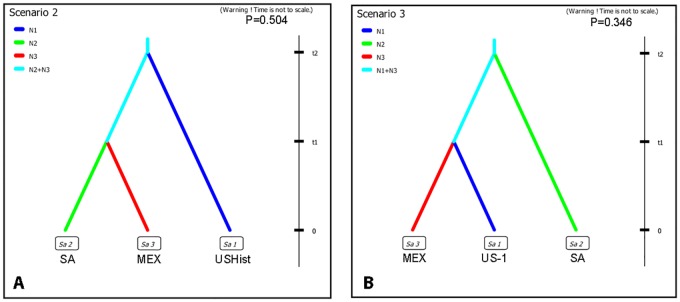
Scenarios for the evolution of *Phytophthora infestans* from sampled populations using Approximate Bayesian Comparison. Posterior probabilities of the top migration scenarios comparing populations from (A) US Historic (USHist), South American (SA) and Mexican (MEX) populations, and (B) US-1, MEX and SA populations. Probabilities are the highest value of a logistic regression of data.

Seven different migration scenarios were examined using SSR allele data and ABC analysis among the USHist, SA, and MEX populations. The scenario with the highest probability (Scenario 2, P = 0.504) was chosen as the most likely model. This scenario was a model in which the USHist lineage diverged first from a common ancestor that then diverged into MEX and SA populations (Fig 6a). Confidence in the scenario choice was evaluated by using simulated datasets to calculate error percentages between the three scenarios with the highest probabilities. Estimation of type I error revealed that 65.6% of simulated datasets using this scenario resulted in the highest posterior probability for Scenario 2 when compared to the two scenarios with the next highest probabilities (Scenarios 3, 5) (type I error, 0.344) (S4 Fig and S10 Table).

We tested the migration scenarios between US-1, SA, and MEX populations and the scenario with the highest probability (Scenario 3, P = 0.346) was a scenario in which SA populations diverged first from a common ancestor followed by divergence of US-1 and MEX populations (Fig 6b). Estimation of type I error indicated that 68.2% of the datasets simulated using Scenario 3 resulted in the highest posterior probability when compared to the scenarios with the next highest probabilities (Scenarios 4 and 1) (type I error, 0.318) (S5 Fig and S10 Table).

## Discussion

### Source of historic US *P*. *infestans*

We examined the population structure of historic late blight using one of the largest globally sourced collection of historic specimens (*n* = 66) and modern samples (*n* = 117) examined to date. Our data revealed the widespread occurrence of a unique historic nuDNA SSR lineage of *P*. *infestans* that we have named the FAM-1 lineage. This lineage was found in historical US specimens collected from 1855 to 1939 (Fig 1) and included specimens reported previously in the FAM phylogenetic group designated by Martin et al. [27]. Interestingly, the oldest known blight-infected herbarium samples from South America (1913, 1939 Columbia; 1942 Costa Rica) also belonged to the FAM-1 SSR lineage. Thus, the FAM-1 lineage was geographically widespread in the US but was also present in Central and South America and persisted for almost 100 years after initial introduction in many geographic areas of the US (Fig 1, blue circles). Our work with the largest set of historic US samples examined to date documents that the same nuclear genome lineage of *P*. *infestans* caused epidemics in both the US and Europe as was suggested by others [23].

Surveys of genotypes to monitor changes in *P*. *infestans* populations are regularly carried out in the US and Europe by USAblight and EUROblight project teams using FTA card sampling [4, 6, 14]. These samples are genotyped using the 12-plex SSR protocol, but baseline data about SSR profiles for early lineages prior to US-1 were missing until now [6]. Documentation of a large number of SSR profiles from historic samples from our work now provides a baseline for comparison to new SSR genotypes and will enable large-scale surveys to determine if/where the historic FAM-1 lineage persists.

Yoshida et al. previously sequenced the HERB-1 nuclear and mitochondrial genomes in eleven historic samples, including ten from Europe and one from North America, and came to the same conclusion that the historic outbreaks in the US and Europe were caused by the same lineage [23]. A second paper by Yoshida et al., which analyzed genomes both from the original work and from those sequenced by Martin et al. [32], brought the total to thirteen genomes and that work also showed a single lineage caused historic US and EU outbreaks [34]. While the HERB-1 mitochondrial and nuclear genomes were initially thought to be extinct [23, 34, 35], work by Martin et al. that involved the sequencing of 44 additional mitogenomes detected the HERB-1 mitochondrial genome in samples of *P*. *infestans* from Mexico and Ecuador from the 1980s and 2000s [33].

The FAM-1 lineage was present in Europe during the first late blight outbreaks (S1 Table). We compared the genetic structure of USHist to EUHist lineages, and show that both New and Old World outbreaks were caused by the same lineage of *P*. *infestans*. Shared allelic diversity was observed between US and EU historic lineages for all values of *K*. Thus, our data suggest that the same FAM-1 lineage caused both the US late blight outbreaks in 1842 and the European outbreaks two years later. Our data also show that the FAM-1 lineage persisted for over 30 years in Europe and with wider examination may likely still be found in present-day European isolates of *P*. *infestans*.

The source of the 19^th^-century late blight outbreaks has been debated [18–20, 25, 30–32, 35]. Our data from multilocus genotyping demonstrate shared allelic diversity between historic lineages in South America, the US, and Europe [27]. Indeed, our current data indicate that US and European famine-era populations share more genetic similarity with the oldest Colombian populations (1913–1929) than the oldest Mexican samples that are known to exist.

Martin et al. [27] used whole genome sequences from a large set of global samples and recently documented that the divergence time of famine-era European lineages occurred before present-day Mexican and South American lineages. Our ABC data corroborate those findings in that the most likely scenario includes the divergence of the FAM-1 lineage before that of more recent SA and MEX lineages. Our data suggest that the FAM-1 lineage emerged in a US metapopulation from either a South or Central American source, spread to Europe to cause famine-era outbreaks, and survived in the New World for a long period of time after that. The present-day SA lineages in our study are likely reintroductions of the pathogen from Mexican sources as others have suggested [65].

The oldest published reports of late blight in SA cited by Neiderhauser are in 1887 from Argentina [18]. However, to our knowledge no 19^th^-century samples exist from either Mexican or South American sources to confirm the 19^th^-century presence of the pathogen in either region. The oldest South American sample in our study was collected from Colombia and belonged to the FAM-1 SSR lineage. Although it would be interesting to examine even older historic samples from South America and Mexico, we have searched herbarium collections and to our knowledge these specimens do not exist. It would be intriguing to sequence and compare the nuclear and mitochondrial genomes of the oldest USHist and Colombian FAM-1 lineages and compare them with those of the EUHist FAM-1 lineages to further elucidate the temporal sequence of historic introductions to the Old World.

Multilocus sequencing of nuclear genes indicated the presence of haplotypes unique to historic populations. In addition, coalescent analyses of two nuclear loci suggest that USHist and EUHist outbreaks arose from a common South American ancestor. Additional historic evidence, such as increased trade in seed potatoes from South America at the time, [3, 25] suggests a more likely scenario in which infected tubers were moved first to the US and then to Europe, providing a potential migration route and source of disease.

Our analyses indicate more migrants moved into historic European populations from US populations than vice versa, suggesting historic US populations of the pathogen contributed to the European outbreaks more than once. Historic literature also suggests that US outbreaks were a source of subsequent EU outbreaks [66]. There are many reports in the 19^th^-century *Gardeners’ Chronicles*, a publication used by farmers and naturalists, describing potential migration routes from shipments of “seed sets” shipped from the US and Canada into Bermuda, the UK, and Europe [66].

Yoshida et al. [23, 34] suggested a single introduction of the ancestral HERB-1 haplotype into Europe from a metapopulation outside of Mexico. While our data support a US metapopulation emergence outside of Mexico, the presence of multiple mitochondrial haplotypes in Europe [33] also suggests multiple introductions of the pathogen may have occurred between 1845 and 1889. It is possible that separate introductions of late blight into Europe may have occurred on infected tubers shipped both directly from the US and from South America [3]. The oldest European sample from 1845 shared a basal lineage with the hybrid species *P*. *andina* from a shared Andean source population [27]. The incongruity of multiple mtDNA haplotypes combined with a single nuclear lineage was observed in Martin et al. [27] with the positioning/clustering of *P*. *andina* samples within the HERB-1 mitochondrial clade. This dichotomy may be the result of long periods of clonal reproduction, with forces such as mitotic recombination and genetic drift creating the variation that results in the observation of multiple subclades that could point towards multiple introductions [27].

### Origin of the US-1 lineage

We and others [23, 31, 35] have suggested that the US-1 lineage emerged later from a metapopulation outside of Mexico. Our data show that the US-1 lineage clearly emerged on a global scale in the mid-20^th^ century and formed a distinct cluster that shares little allelic diversity with either the US or EU historic FAM-1 lineage. Thus, the US-1 lineage is not a direct descendent of famine-era outbreaks, but is a sister lineage, as is suggested by both mitochondrial and nuclear phylogenies done with whole genome datasets [23, 33, 34, 67, 68]. It is possible that US-1 and the FAM-1 lineages may have come from different sources. Martin et al. [27] suggested US-1 and famine-era lineages may have originated on non-*S*. *tuberosum* hosts. It is possible that the two lineages diverged from a common ancestor on two different host species, resulting in the apparent differences in allelic diversity. US-1 could have been introduced into the US from a different source and then displaced the FAM-1 lineage. However, Martin et al. [27] noted that relationships inferred from phylogenetic analysis of the *P*. *infestans* mitogenome may be inconsistent with relationships observed in the nuclear genome, and a more comprehensive examination of samples would be needed to discern potential sources. Mitochondrial phylogenies support the contention that type Ib (US-1) and type Ia mitochondrial lineages are sister lineages [67, 68]. Coalescent analysis indicates that the oldest mutations that gave rise to US-1 populations arose in South America [27, 33]. We also identified several recent SA isolates of *P*. *infestans* from Peru that were clustered with the US-1 lineage. Herbarium samples from the US placed the US-1 lineage in Texas in 1931 (S1 Table). Our migration analyses indicated migration of US-1 into both Mexico and South America, but greater numbers of migrants moved into Mexico. Thus, it is possible that the US-1 emerged from a South American source. The rare occurrence of the US-1 lineage in Mexico suggests it may be a recent immigrant there [7, 33, 69, 70]. Sequencing additional US-1 samples from Mexico would help clarify this question.

The increasing frequency of the US-1 lineage in the WWII era is intriguing. The US-1 lineage was used in Cold War era research at Fort Detrick to test for virulence on potato germplasm [71]. In fact, historic isolates used in those studies were examined in our work here, (Mannon Gallegly, pers. comm.), and one of those isolates belonged to the US-1 lineage. How US-1 became so widely dispersed in the mid-20^th^ century is unknown [7], but since US-1 is sensitive to metalaxyl, it was displaced by other fungicide-resistant lineages of *P*. *infestans* when this compound was deployed in the late 1970s [70]. The introduction of new migrants from Mexico subsequently displaced the US populations of the US-1 lineage in the early 1990s [70].

### Recent migrations from Mexico of aggressive lineages

The oldest Mexican herbarium voucher infected with late blight examined in our study, collected by John Neiderhauser in Chihuahua in 1948, was not assigned to the FAM-1 lineage, but was more similar to modern Mexican and US aggressive lineages. Mexican lineages clustered with many recent lineages of *P*. *infestans* in the US. Admixture between Mexican populations and modern US lineages, especially US-6, US-7, and US-8 lineages, support the theory of Fry and others [6, 70] that current populations of *P*. *infestans* in the US are primarily the result of migrations out of Mexico. Ancestral recombination graphs clearly documented the recombinant nature of one aggressive lineage, US-11, a clonal lineage that has been observed in recent years on tomato in the US [6, 15].

The cause of the apparent displacement of the historic-era populations of *P*. *infestans* after the 1940s is unknown at this time (Fig 3). Clearly there was an increase in potato breeding programs and movement of potato germplasm and the pathogen worldwide during and after WWII [71]. Potatoes were also used as a valuable food source to feed both the Allied and German troops during WWII. Since historic FAM lineages lack many of the genes responsible for virulence on modern potatoes, this could have led to its decline [23, 32]. Modern populations of *P*. *infestans* have migrated from Mexico on infected potatoes on multiple occasions, and genotype shifts have been observed [14, 70]. The most recent population shift has been the displacement of the US-22 lineage by the US-23 lineage in the US over the past 5 years [13, 14, 15]. A similar observation has been made in Europe, where 13_A2, noted for its aggressive virulence, has become the dominant lineage [4]. These shifts presumably indicate the replacement of older lineages by newer lineages with higher fitness [15]. These lineages may be more aggressive, able to cause disease on both tomato and potato, or be resistant to fungicides

While our evidence suggests a South American origin for the FAM-1 lineage of *P*. *infestans*, to fully understand the evolutionary history of the pathogen in the New World, more samples are needed [72, 73]. Unfortunately, historic samples available for study from Mexico and South America are limited, particularly older herbarium samples. It is problematic that these samples do not exist, but further sampling of modern late blight from the Andean region of South America, especially from wild *Solanum* species, would improve our ability to draw inferences about FAM-1’s origins.

Recently, we suggested that it is possible that late blight was introduced into Mexico on a non-potato host [27], and that additional sampling from other Solanaceous hosts could help elucidate the role of host jumps in the evolution of the pathogen [1,74]. Goss et al. [65] suggested a similar non-potato origin for *P*. *infestans* as a species, emphasizing the need to collect from hosts outside of potato and tomato crops. Our data document that the FAM-1 lineage occurred on several wild species early after its introduction into Europe on *Anthocercis ilicifolia* Hook., *Solanum dulcamara* L., *Solanum nigrum* L., and *Petunia hybrida* E. Vilm. and in the Americas (S1 Table) [8, 9, 29]. It is possible that the metapopulation source of *P*. *infestans* alluded to by others [23, 27] may be discerned by a more thorough examination of wild hosts. In addition, previous studies have indicated passage through a wild host may increase aggressiveness of *P*. *infestans*, and this could have ecological implications for the control of the disease [75]. *P*. *infestans* may have migrated from a wild *Solanum* host to domesticated potato and vice versa more than once [18, 19, 27]. There is evidence from herbarium records that non-*S*. *tuberosum* hosts played a role in the dispersal of this pathogen in the recent past [27, 29]. Thus, further studies to understand the role of host biodiversity and the movement of wild species in migrations of *Phytophthora infestans* are needed.

## Supporting Information

S1 Fig Ancestral recombination graph (ARG) of sequences from the *ras* locus from modern and historic herbarium collections of *Phytophthora infestans*. Green and yellow dots indicate points of coalescence. Recombination events are indicated by blue circles. Numbers within circles indicate the position of the site before recombination takes place (see S4 Table). Numbers along branches indicate the number of mutations between points. P: The origin of the prefix contribution to the recombination event; S: The origin of the suffix contribution to the recombination event.
(TIF)

S2 Fig Ancestral recombination graph (ARG) of sequences from the *PiAVR2* locus from modern and historic herbarium collections of *Phytophthora infestans*. Yellow dot indicates the point of coalescence. Numbers along branches indicate the number of mutations between points.
(TIF)

S3 Fig Ancestral recombination graph (ARG) of sequences from the P3 mitochondrial region from modern and historic herbarium collections of *Phytophthora infestans*. Yellow and green dots indicate the points of coalescence. Numbers along branches indicate the number of mutations between points.
(TIF)

S4 Fig Posterior probabilities of migration scenarios involving populations of *Phytophthora infestans* from South America (SA), Mexico (MEX), and famine era US (USHist). Probabilities are based on the highest value of a logistic regression of data.
(TIF)

S5 Fig Posterior probabilities of migration scenarios involving populations of *Phytophthora infestans* from South America (SA), Mexico (MEX), and Ib haplotypes (US1). Probabilities are based on the highest value of a logistic regression of data.
(TIF)

S1 Table Sample number, date of collection, location, genotype or mitochondrial lineage, group and host of isolates of *Phytophthora infestans* from herbarium specimens and modern collections used in this study. (DOCX)

S2 Table Target gene region, primer name, primer sequence, primer size, location of primer on target DNA, and reference source of PCR primers used in the study. (DOCX)

S3 Table Prior distributions for Do It Yourself Approximate Baysian Computation (DIYABC) scenarios. Summary statistics for all runs: within population statistics: mean number of alleles and mean genetic diversity; between sample statistics: mean number of alleles, mean genetic diversity, Fst, shared allele distance, (δμ) ^2^, and maximum likelihood coefficient of admixture.
(DOCX)

S4 Table Diversity statistics for clone corrected microsatellite data for all 12 loci within populations of *Phytophthora infestans*. (DOCX)

S5 Table Population statistics, diversity indices, and neutrality tests among *Phytophthora infestans* populations using *ras*, *PiAVR2*, and the P3 mitochondrial region. (DOCX)

S6 Table Distribution of haplotypes and base substitution events in nuclear and mitochondrial genes of *Phytophthora infestans*. (DOCX)

S7 Table Population subdivision of *Phytophthora infestans* populations at *ras* locus according to Hudson’s test statistics Ks (upper right matrix) and Kst (lower left matrix). (DOCX)

S8 Table Locus, haplotype, isolate identities and population sampled for modern and historic isolates of *Phytophthora infestans*. (DOCX)

S9 Table Isolation model (IM) parameter estimates for *Phytophthora infestans* populations from US Historic (USHist), EU Historic (EUHist), Mexican (MEX), US-1 and South America (SA) based on variation in the *ras* or *PiAVR2* nuclear loci. (DOCX)

S10 Table Posterior probabilities and confidence intervals from three *Phytophthora infe*stans populations. Probabilities listed are the highest values as calculated over a 10-sample logistic regression.
(DOCX)
