# Supplementary figures and images for: Historic Late Blight Outbreaks Caused by a Widespread Dominant Lineage of Phytophthora infestans (Mont.) de Bary
Source: PLoS One. 2016 Dec 28;11(12):e0168381. doi: 10.1371/journal.pone.0168381 (PMC5193357; doi:10.1371/journal.pone.0168381)

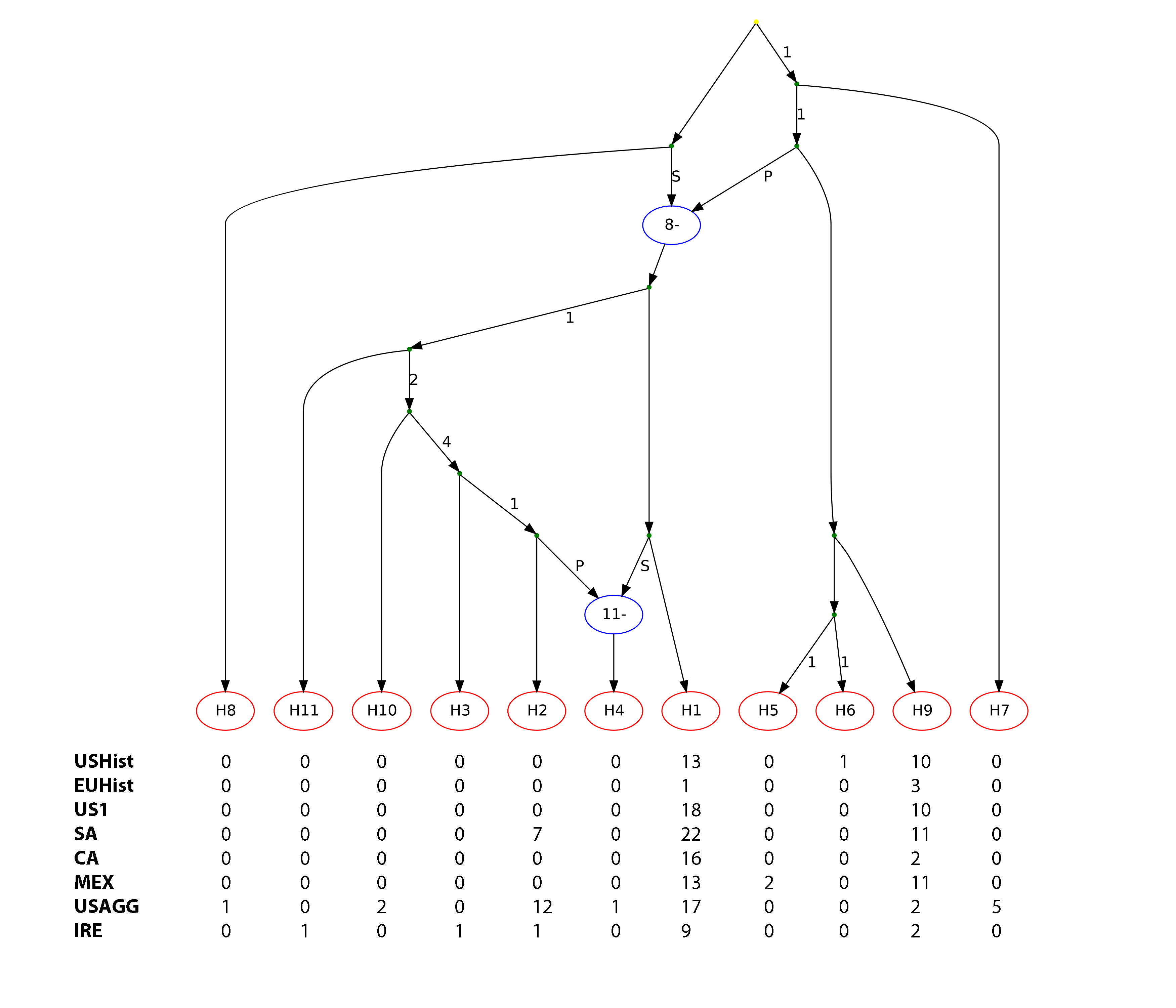

Supplement: S1 Fig — Green and yellow dots indicate points of coalescence. Recombination events are indicated by blue circles. Numbers within circles indicate the position of the site before recombination takes place (see S4 Table). Numbers along branches indicate the number of mutations between points. P: The origin of the prefix contribution to the recombination event; S: The origin of the suffix contribution to the recombination event. (TIF) [file pone.0168381.s001.tif]

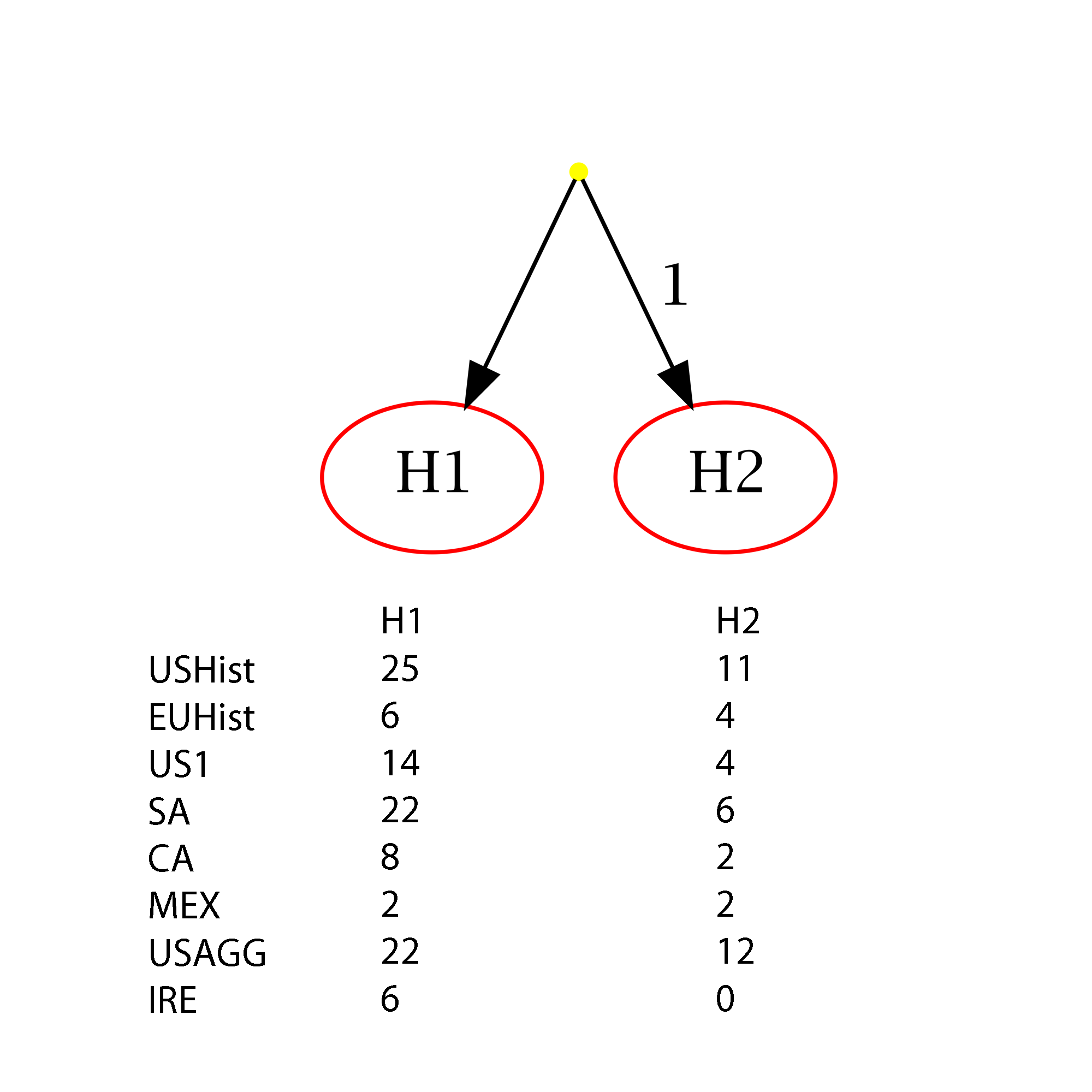

Supplement: S2 Fig — Yellow dot indicates the point of coalescence. Numbers along branches indicate the number of mutations between points. (TIF) [file pone.0168381.s002.tif]

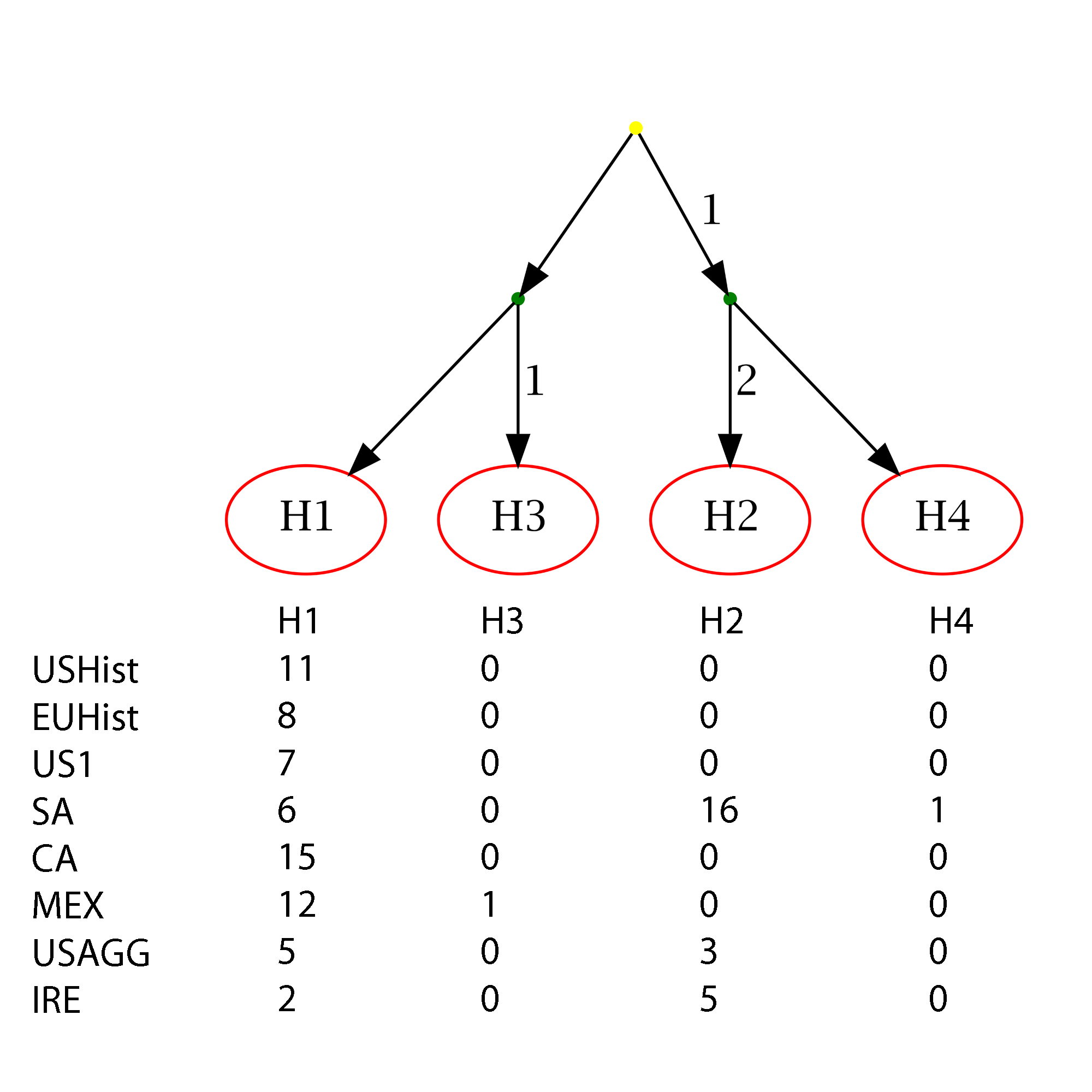

Supplement: S3 Fig — Yellow and green dots indicate the points of coalescence. Numbers along branches indicate the number of mutations between points. (TIF) [file pone.0168381.s003.tif]

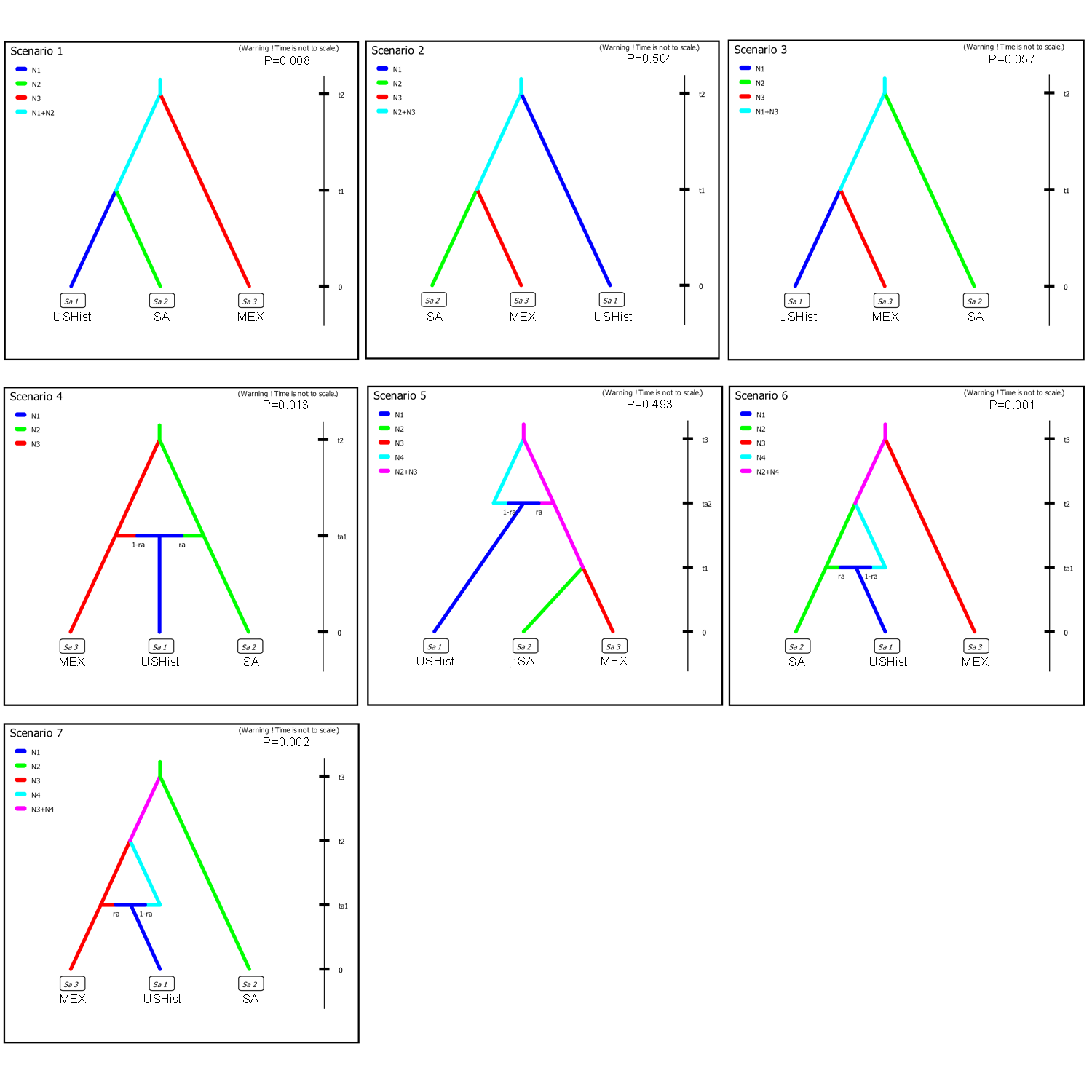

Supplement: S4 Fig — Probabilities are based on the highest value of a logistic regression of data. (TIF) [file pone.0168381.s004.tif]

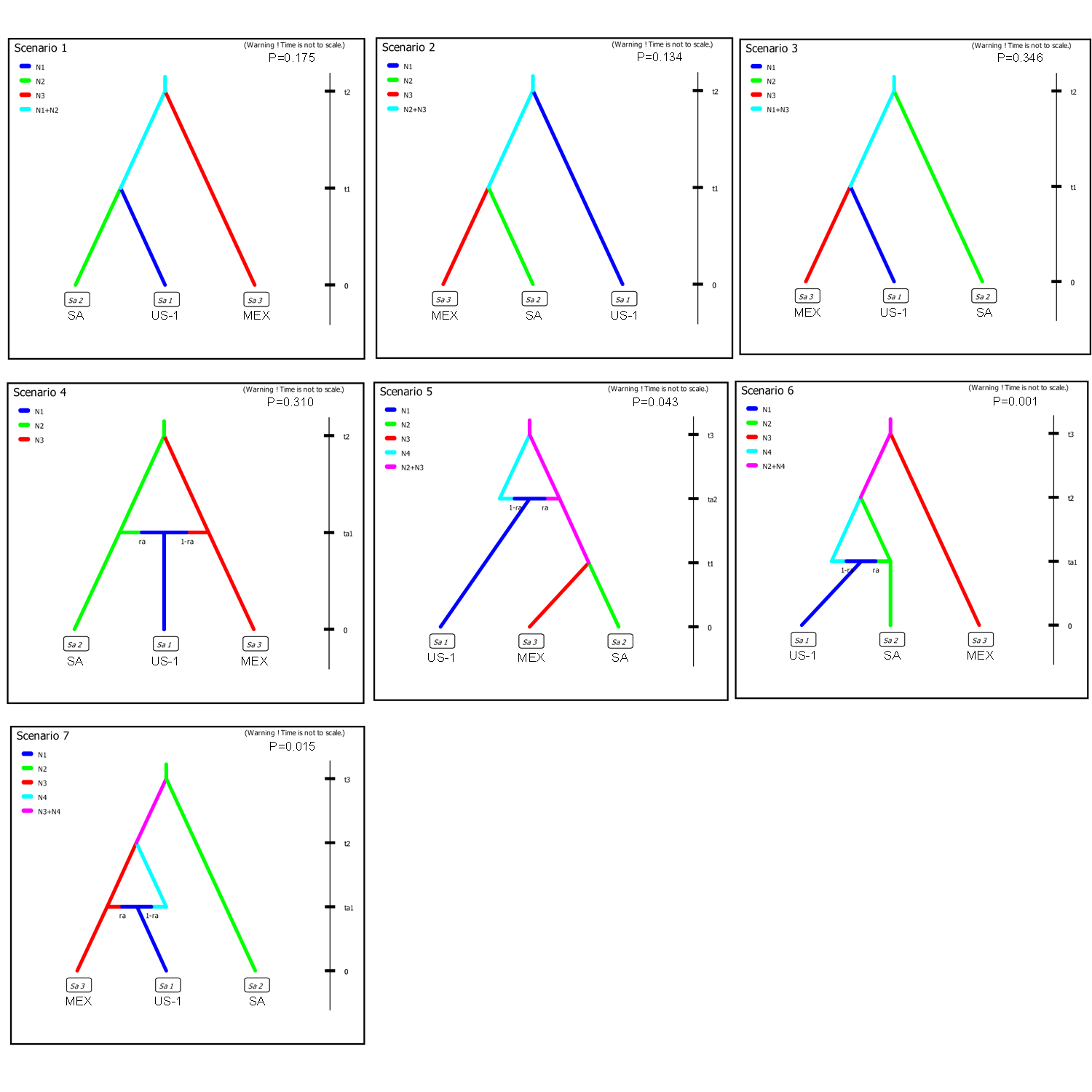

Supplement: S5 Fig — Probabilities are based on the highest value of a logistic regression of data. (TIF) [file pone.0168381.s005.tif]
