# Supplementary material for: Historic Late Blight Outbreaks Caused by a Widespread Dominant Lineage of Phytophthora infestans (Mont.) de Bary
Source: PLoS One. 2016 Dec 28;11(12):e0168381. doi: 10.1371/journal.pone.0168381 (PMC5193357; doi:10.1371/journal.pone.0168381)
Supplement: S1 Table — (DOCX) [file pone.0168381.s006.docx]

**S1 Table**. **Sample number, date of collection, location, genotype or mitochondrial lineage, group and host of isolates of *Phytophthora infestans* from herbarium specimens and modern collections used in this study.**

| **Sample Number** | **Date** | **Country** | **Genotype/mtDNA haplotype** | **Group** | **Host** | **Collector/Source** |
| --- | --- | --- | --- | --- | --- | --- |
| **Herbarium Samples ^a^** |  |  |  |  |  |  |
| K 7 | 1846 | Ireland | FAM-1^b^/HERB-1 | EUHist | *Anthocercis ilicifolia* | D. Moore |
| BPI US0186686 | 1855 | US (NY) | ―/HERB-1 | USHist | Potato | J. B. Ellis |
| FH 219 | 1873 | Germany | FAM-1/HERB-1 | EUHist | Tomato | F. de Thumen |
| K 22 | 1875 | Britain | FAM-1/HERB-1 | EUHist | Potato | J. E. Vize |
| UPS 1 | 1876 | Denmark | FAM-1/HERB-1 | EUHist | Potato | E. Rostrup |
| FH 222 | 1877 | Germany | FAM-1/HERB-1 | EUHist | Potato | P. Magnus |
| DBN 11 | 1878 | England | FAM-1/HERB-1 | EUHist | Potato | Unknown |
| K 43 | 1879 | Britain | FAM-1/Type I | EUHist | Potato | M. J. Berkeley |
| BPI US0186680 | 1880 | US (WI) | FAM-1/HERB-1 | USHist | Potato | W. Trelease |
| BPI US0186932 | 1882 | US (ME) | FAM-1/HERB-1 | USHist | Potato | F. L. Harvey |
| FH 288 | 1882 | US (IL) | FAM-1/HERB-1 | USHist | Potato | A. B. Seymour |
| BPI US0186961 | 1882 | US (WI) | FAM-1/HERB-1 | USHist | Potato | G. A. Pabodie |
| UPS 2 | 1882 | Sweden | FAM-1/HERB-1 | EUHist | Potato | J. Eriksson |
| FH 263 | 1882 | Hungary | FAM-1/Not Ib | EUHist | Potato | Linhardt |
| BPI US0186904 | 1885 | US (NY) | FAM-1/HERB-1 | USHist | Potato | W. R. Dudley |
| BPI US0186905 | 1889 | US (NY) | FAM-1/HERB-1 | USHist | Potato | W. R. Dudley |
| BPI US0186656 | 1889 | US (MA) | FAM-1/HERB-1 | USHist | Potato | W. C. Sturgis |
| BPI US0186913 | 1889 | US (MA) | FAM-1/HERB-1 | USHist | Potato | J. E. Humphrey |
| BPI 796293 | 1889 | US (NJ) | FAM-1/HERB-1 | USHist | Potato | J. B. Ellis |
| K 79 | 1889 | Germany | FAM-1/HERB-1 | EUHist | Potato | P. Hennings |
| FH 206 | 1891 | US (NC) | FAM-1/HERB-1 | USHist | Potato | A. B. Seymour |
| BPI US0186674 | 1891 | US (MD) | FAM-1/HERB-1 | USHist | Potato | W. T. Swingle |
| BPI US0186920 | 1891 | US (NY) | FAM-1/HERB-1 | USHist | Potato | M. B. Thomas |
| **Sample Number** | **Date** | **Country** | **Genotype/mtDNA haplotype** | **Group** | **Host** | **Collector/Source** |
| BPI US0186996 | 1895 | US (VT) | FAM-1/HERB-1 | USHist | Potato | L. R. Jones |
| BPI US0186668 | 1895 | US (TN) | FAM-1/HERB-1 | USHist | Potato | F. L. Scribner |
| BPI US0186964 | 1896 | US (NY) | FAM-1/Not Ib | USHist | Potato | B. M. Duggar |
| BPI US0186897 | 1896 | US (VT) | FAM-1/HERB-1 | USHist | Potato | L. R. Jones |
| BPI US0186900 | 1898 | US (VT) | FAM-1/Not Ib | USHist | Potato | L. R. Jones, Orton |
| BPI US0186835 | 1898 | Italy | FAM-1/HERB-1 | EUHist | *Solanum dulcamara* | T. Ferraris |
| BPI US0186891 | 1902 | US (CT) | FAM-1/HERB-1 | USHist | Potato | G. P. Clinton |
| BPI US0186882 | 1902 | US (CT) | FAM-1/Not Ib | USHist | Potato | G. P. Clinton |
| UPS 9 | 1905 | Sweden | FAM-1/HERB-1 | EUHist | Potato | J. Eriksson |
| BPI US0186890 | 1906 | US (CT) | FAM-1/HERB-1 | USHist | Potato | G. P. Clinton |
| BPI US0186987 | 1907 | US (ME) | FAM-1/HERB-1 | USHist | Potato | W. J. Morse |
| BPI US0186967 | 1907 | US (ME) | FAM-1/HERB-1 | USHist | Potato | M. J. Morse |
| FH 283 | 1912 | US (NY) | FAM-1/HERB-1 | USHist | Potato | C. Chupp |
| BPI US0186968 | 1913 | Colombia | FAM-1/HERB-1 | SA | Potato | J. M. Vargas Vergara |
| BPI US0186979 | 1915 | US (PA) | FAM-1/HERB-1 | USHist | Potato | G. R. Lyman |
| BPI US0186868 | 1918 | US (CT) | FAM-1/HERB-1 | USHist | Potato | G. P. Clinton |
| FLAS 712 | 1923 | US (FL) | FAM-1/HERB-1 | USHist | Potato | Weber |
| BPI US0186872 | 1928 | US (CT) | FAM-1/HERB-1 | USHist | Potato | G. P. Clinton |
| FH 292 | 1929 | Colombia | FAM-1/HERB-1 | SA | Potato | C. H. Chandon |
| BPI 796306 | 1930 | US (WV) | FAM-1/HERB-1 | USHist | Potato | W. A. Orton |
| BPI 796307 | 1930 | US (WV) | FAM-1/HERB-1 | USHist | Tomato | W. A. Orton |
| BPI US0186927 | 1931 | US (TX) | US-1/Ib | US1 | Potato | W. J. Bach |
| BPI US0187006 | 1931 | US (TX) | US-1/Ib | US1 | Potato | A. A. Stalmach |
| BPI US0186928 | 1934 | US (AK) | FAM-1/HERB-1 | USHist | Potato | G. F. Gravatt |
| **Sample Number** | **Date** | **Country** | **Genotype/mtDNA haplotype** | **Group** | **Host** | **Collector/Source** |
| BPI US0186841 | 1937 | US (OR) | FAM-1/HERB-1 | USHist | *S. nigrum* | R. Sprague |
| DBN 2 | 1937 | Ireland | FAM-1/HERB-1 | EUHist | Potato | P. O. O’Connor |
| BPI US0187016 | 1938 | US (FL) | FAM-1/HERB-1 | USHist | Potato | H. A. Edson |
| BPI US0186954 | 1939 | US (WA) | FAM-1/IIa | USHist | Potato | L. Campbell |
| BPI US0186816 | 1941 | Guatemala | ―/HERB-1 | CA | Tomato | A. S. Muller |
| BPI US0187022 | 1942 | Costa Rica | FAM-1/HERB-1 | CA | Potato | R. Mendez |
| BPI US0186832 | 1942 | Guatemala | ―/HERB-1 | CA | *Petunia hibrida* | J. A. Stevenson/ Muller |
| BPI US0186661 | 1946 | US (CT) | US-1/Ib | US1 | Tomato | A. D. McDonnell |
| FH 287 | 1948 | Canada | US-1/Ib | US1 | Potato | B. O. Saville |
| BPI US0186972 | 1948 | Mexico | ―/IIa | MEX | Potato | J. S. Niederhauser |
| CUP 4 | 1950 | US (MN) | US-1/Ib | US1 | Potato | D. Thurston |
| PA222 | 1950-1960 | US (PA) | US-1/Ib | US1 | ― | M. Gallegly |
| K 126 | 1952 | Britain | US-1/Ib | US1 | Potato | J. H. H. |
| BPI US0186956 | 1954 | Nicaragua | ―/Ia | CA | Potato | S. C. Litzenberger |
| BPI US0186953 | 1956 | Nicaragua | ―/IIb | CA | Potato | S. C. Litzenberger |
| BPI US0186807 | 1958 | US (OH) | US-1/Ib | US1 | Tomato | J. L. Cunningham |
| P445 | 1966 | Mexico | ―/HERB-1 | MEX | ― | M. Gallegly |
| BPI US0186908 | 1967 | Ecuador | US-1/Ib | US1 | Potato | V. C. Withee |
| K 125 | 1970 | Britain | US-1/Ib | US1 | Potato | R. Dennis |
|  |  |  |  |  |  |  |
| **Modern isolates** |  |  |  |  |  |  |
| 94-1 | 1994 | US (NC) | US-1/Ib | US1 | Potato | J. Ristaino |
| 95-6 | 1995 | US (PA) | US-1/Ib | US1 | Potato | B. Christ |
| US920141 | 1992 | US (ND) | US-1/Ib | US1 | Potato | W. Fry |
| 188.1.1 | 1994 | Canada | US-1/Ib | US1 | Potato | Z. Punja |
| 920159 | 1992 | US (WA/OR) | US-6/IIb | USAGG | ― | W. Fry |
| **Sample Number** | **Date** | **Country** | **Genotype/mtDNA haplotype** | **Group** | **Host** | **Collector/Source** |
| 94-55 | 1994 | US (NY) | US-6/IIb | USAGG | Tomato | W. Fry |
| 94-52 | 1994 | US (NY) | US-6/IIb | USAGG | Potato | W. Fry |
| 94-22 | 1994 | US (NC) | US-7/Ia | USAGG | Tomato | J. Ristaino |
| 94-11-2 | 1994 | US (NC) | US-7/Ia | USAGG | Tomato | J. Ristaino |
| 93-3 | 1993 | US (NC) | US-7/Ia | USAGG | Tomato | J. Ristaino |
| 94-53 | 1994 | US (NC) | US-7/Ia | USAGG | Potato | J. Ristaino |
| 2.1.3 | 1993 | Canada | US-7/Ia | USAGG | Potato | Z. Punja |
| 98-97 | 1998 | US (NC) | US-8/Ia | USAGG | Potato | J. Ristaino |
| RS2009P1 | 2009 | US (PA) | US-8/Ia | USAGG | Potato | B. Gugino |
| 98-82 | 1998 | US (NC) | US-8/Ia | USAGG | Potato | J. Ristaino |
| 94-8-4 | 1994 | US (NC) | US-8/Ia | USAGG | Potato | J. Ristaino |
| VA09-pot | 2009 | US (VA) | US-8/Ia | USAGG | Potato | J. Ristaino |
| 97-24 | 1997 | US (NC) | US-8/Ia | USAGG | Potato | J. Ristaino |
| 342.1.1 | 1995 | Canada | US-11/IIb | USAGG | ― | Z. Punja |
| 268.1.5 | 1995 | Canada | US-11/IIb | USAGG | ― | Z. Punja |
| US980059 | 1998 | US (AK) | US-11/IIb | USAGG | Potato | W. Fry |
| US940478 | 1994 | US (WA) | US-11/IIb | USAGG | ― | W. Fry |
| US980042 | 1998 | US (CA) | US-11/IIb | USAGG | Tomato | W. Fry |
| TN-070-A | 2007 | US (TN) | US-22/Ia | USAGG | Tomato | K. Deahl |
| TNFL-2 | 2007 | US (TN) | US-22/Ia | USAGG | Tomato | K. Deahl |
| NY09- | 2009 | US (NY) | US-22/Ia | USAGG | Tomato | M. McGrath |
| BL2009P4 | 2009 | US (PA) | US-23/Ia | USAGG | Potato | B. Gugino |
| PSUTomA | 2009 | US (PA) | US-23/Ia | USAGG | Tomato | B. Gugino |
| 63EB | 2014 | US (NC) | US-23/Ia | USAGG | Tomato | R. Gardner |
| NC870 | 2014 | US (NC) | US-23/Ia | USAGG | Tomato | R. Gardner |
| NC1CELBR | 2014 | US (NC) | US-23/Ia | USAGG | Tomato | R. Gardner |
| ND884-1 | 2009 | US (ND) | US-24/Ia | USAGG | Potato | G. Secor |
| ND888 | 2009 | US (ND) | US-24/Ia | USAGG | Potato | G. Secor |
| ND10-936 | 2010 | US (ND) | US-24/Ia | USAGG | Potato | G. Secor |
| BOL3 | ― | Bolivia | BR-1/IIa | SA | Potato | PROINPA |
| BOL9 | ― | Bolivia | BR-1/IIa | SA | Potato | PROINPA |
| B217 | 1998 | Brazil | BR-1/IIa | SA | Potato | E. Mizubuti |
| B219 | 1998 | Brazil | BR-1/IIa | SA | Potato | E. Mizubuti |
| B189 | 1998 | Brazil | BR-1/IIa | SA | Potato | E. Mizubuti |
| B193 | 1998 | Brazil | BR-1/IIa | SA | Potato | E. Mizubuti |
| DR-4 | 2001 | Costa Rica | CR-1/Ia | CA | Potato | A. Brenes |
| ZB | ― | Costa Rica | CR-1/Ia | CA | Potato | A. Brenes |
| **Sample Number** | **Date** | **Country** | **Genotype/mtDNA haplotype** | **Group** | **Host** | **Collector/Source** |
| ZE | 2001 | Costa Rica | CR-1/Ia | CA | Potato | A. Brenes |
| 52 | 2003 | Costa Rica | CR-1/Ia | CA | *S. longiconicum* | A. Brenes |
| 61 | 2003 | Costa Rica | CR-1/Ia | CA | Potato | A. Brenes |
| 141 | 2003 | Costa Rica | CR-1/Ia | CA | Potato | A. Brenes |
| CI | 2000 | Costa Rica | CR-1/Ia | CA | Potato | A. Brenes |
| 152 | 2003 | Costa Rica | CR-1/Ia | CA | Potato | A. Brenes |
| 42 | ― | Costa Rica | CR-1/Ia | CA | ― | J. Ristaino |
| 92 | ― | Costa Rica | CR-1/Ia | CA | Potato | J. Ristaino |
| 201 | ― | Costa Rica | CR-1/Ia | CA | Potato | J. Ristaino |
| 211 | ― | Costa Rica | CR-1/Ia | CA | Potato | J. Ristaino |
| 71 | ― | Costa Rica | CR-1/Ia | CA | Potato | J. Ristaino |
| 221 | ― | Costa Rica | CR-1/Ia | CA | Potato | J. Ristaino |
| 81 | ― | Costa Rica | CR-1/Ia | CA | Potato | J. Ristaino |
| 231 | ― | Costa Rica | CR-1/Ia | CA | ― | J. Ristaino |
| 91 | ― | Costa Rica | CR-1/Ia | CA | Potato | J. Ristaino |
| 151 | 2003 | Costa Rica | CR-1/Ia | CA | Potato | A. Brenes |
| EC3092 | 1997 | Ecuador | EC-1/IIa | SA | *S. phureja* | G. Forbes |
| EC3094 | 1997 | Ecuador | EC-1/IIa | SA | *S. phureja* | G. Forbes |
| EC3199 | 1998 | Ecuador | EC-1/IIa | SA | *S. tuquerense* | G. Forbes |
| EC3253 | 1999 | Ecuador | EC-1/IIa | SA | *S. columbianum* | G. Forbes |
| EC3154 | 1998 | Ecuador | EC-1/IIa | SA | *S. andreanum* | G. Forbes |
| EC3298 | 2000 | Ecuador | EC-1/IIa | SA | *S. tetrapetalum* | G. Forbes |
| EC3300 | 2000 | Ecuador | EC-1/IIa | SA | *S. paucijugum* | G. Forbes |
| 6/95 | 1995 | Ireland | ND/IIa | IRE | Potato | L. Cooke |
| 31/95 | 1995 | Ireland | ND/IIa | IRE | Potato | L. Cooke |
| 3/99 | 1999 | Ireland | V-3/IIa | IRE | Potato | L. Cooke |
| 21A/93 | 1993 | Ireland | ND/IIa | IRE | Potato | L. Cooke |
| 15/99 | 1999 | Ireland | IE-1/IIa | IRE | Potato | L. Cooke |
| 12/94 | 1994 | Ireland | ND/Ia | IRE | Potato | L. Cooke |
| 16/99 | 1999 | Ireland | V-1/Ia | IRE | Potato | L. Cooke |
| 15/94 | 1994 | Ireland | IE-3/Ia | IRE | Potato | L. Cooke |
| 18/94 | 1994 | Ireland | ND/IIa | IRE | Potato | L. Cooke |
| PIC97180 | 1997 | Mexico | ND/Ia | MEX | Potato | N. Grünwald |
| PIC97630 | 1997 | Mexico | ND/Ia | MEX | Potato | N. Grünwald |
| **Sample Number** | **Date** | **Country** | **Genotype/mtDNA haplotype** | **Group** | **Host** | **Collector/Source** |
| PIC98392 | 1998 | Mexico | ND/Ia | MEX | *S. demissum* | N. Grünwald |
| PIC97207 | 1997 | Mexico | ND/Ia | MEX | Potato | N. Grünwald |
| PIC97652 | 1997 | Mexico | ND/Ia | MEX | Potato | N. Grünwald |
| PIC97224 | 1997 | Mexico | ND/Ia | MEX | Potato | N. Grünwald |
| PIC98301 | 1998 | Mexico | ND/Ia | MEX | Potato | N. Grünwald |
| PIC98305 | 1998 | Mexico | ND/Ia | MEX | Potato | N. Grünwald |
| PIC98366 | 1998 | Mexico | ND/Ia | MEX | *S. demissum* | N. Grünwald |
| PIC97388 | 1997 | Mexico | ND/Ia | MEX | Potato | N. Grünwald |
| PIC98369 | 1998 | Mexico | ND/Ia | MEX | *S. demissum* | N. Grünwald |
| PIC97605 | 1997 | Mexico | ND/Ia | MEX | Potato | N. Grünwald |
| PIC98372 | 1998 | Mexico | ND/Ia | MEX | *S. demissum* | N. Grünwald |
| PIC97620 | 1997 | Mexico | ND/Ia | MEX | Potato | N. Grünwald |
| PIC98388 | 1998 | Mexico | ND/Ia | MEX | *S. demissum* | N. Grünwald |
| PIC97022 | 1997 | Mexico | ND/HERB-1 | MEX | Potato | N. Grünwald |
| PIC97349 | 1997 | Mexico | ND/Ia | MEX | Potato | N. Grünwald |
| PIC98382 | 1998 | Mexico | ND/Ia | MEX | Potato | N. Grünwald |
| PIC97370 | 1997 | Mexico | ND/Ia | MEX | Potato | N. Grünwald |
| PIC97323 | 1997 | Mexico | ND/Ia | MEX | Potato | N. Grünwald |
| PIC97316 | 1997 | Mexico | ND/Ia | MEX | Potato | N. Grünwald |
| PIC97229 | 1997 | Mexico | ND/Ia | MEX | Potato | N. Grünwald |
| PCZ026 | 1997 | Peru | PE-6/IIa | SA | Potato | W. Pérez |
| PPU003 | 1997 | Peru | ND/IIa | SA | Potato | W. Pérez |
| PCZ033 | 1997 | Peru | EC1.1/IIa | SA | Potato | W. Pérez |
| PER802 | 1985 | Peru | US-1/Ib | US1 | Potato | P. Tooley |
| PCZ098 | 1997 | Peru | EC1.2/IIa | SA | Potato | R. Nelson |
| PCZ118 | 1997 | Peru | EC1.2/IIa | SA | Potato | W. Pérez |
| PHU006 | 1996 | Peru | EC-1/IIa | SA | Potato | R. Morales |
| PER832 | 1986 | Peru | US-1/Ib | US1 | Potato | P. Tooley |
| POX004 | 1997 | Peru | EC-1/IIa | SA | Potato | R. Nelson |
| PCZ007 | 1997 | Peru | PE-3/Ia | SA | Potato | W. Pérez |
| PCO038 | 1997 | Peru | EC-1/IIa | SA | Potato | W. Pérez |
| PCZ050 | 1997 | Peru | PE-3/Ia | SA | Potato | M. Coca |
| PPA008 | 1998 | Peru | ND/IIa | SA | Potato | E. de la Torre |
| PVM004 | 1998 | Peru | ND/IIa | SA | Potato | W. Pérez |
| PER810 | 2008 | Peru | US-1/Ib | US1 | *S. chiquidenum* | P. Tooley |
| PCA014 | 1999 | Peru | US-1/Ib | US1 | Potato | G. Garry |
| **Sample Number** | **Date** | **Country** | **Genotype/mtDNA haplotype** | **Group** | **Host** | **Collector/Source** |
| PLL018 | 2000 | Peru | EC-1/IIa | SA | Potato | A. Salas |
| PER803 | 2008 | Peru | US-1/Ib | US1 | Potato | P. Tooley |
| PCA001 | 1999 | Peru | PE-3/Ia | SA | Potato | G. Garry |
| PER809 | 2009 | Peru | US-1/Ib | US1 | *S. plurae* | P. Tooley |
| PAN002 | 1999 | Peru | EC-1/IIa | SA | *S. urophylum* | A. Salas |

^a^ Prefix indicates the herbarium code for the source of the material.

^b^ The SSR alleles for the FAM-1 genotype are: 161/200, 268/268, 341/356, 106/141, 267/267, 288/294, 171/175, 191/191, 246/246, 274/274, 173/173, 211/215 for the PiG11, Pi02, PinfSSR11, D13, PinfSSR8, PinfSSR4, Pi04, Pi70, PinfSSR6, Pi63, PinfSSR2, and Pi4B loci, respectively. Alleles displayed are based on a visual consensus of the samples analyzed in this study. Slight variation in length between samples is expected.
