# Supplementary material for: Historic Late Blight Outbreaks Caused by a Widespread Dominant Lineage of Phytophthora infestans (Mont.) de Bary
Source: PLoS One. 2016 Dec 28;11(12):e0168381. doi: 10.1371/journal.pone.0168381 (PMC5193357; doi:10.1371/journal.pone.0168381)
Supplement: S2 Table — (DOCX) [file pone.0168381.s007.docx]

**S2 Table. Target gene region, primer name, primer sequence, primer size, location of primer on target DNA, and reference source of PCR primers used in the study.**

| **Target Region** | **Primer Name** | **Primer Sequence** | **Size** | **Location of Primer^a^** | **Source** |
| --- | --- | --- | --- | --- | --- |
| *Intron Ras* | IRF | 5` TTGCAGCACAACCCAAGACG 3` | 20 | 442 – 461 | Chen and Roxby, 1996 |
|  | IRR | 5` TGCACGTACTATTCGGGGTTC 3` | 21 | 768 – 789 | Chen and Roxby, 1996 |
|  | IRF1^b^ | 5’ TCTCGGTGTAGGTGCCTTTC 3’ | 20 | 492 – 511 | This report |
|  |  |  |  |  |  |
| *ras* | RASF | 5` CGTGTCTGCTTCTCCGTTTCG 3` | 21 | 916 – 936 | Ristaino *et al*. 2001 |
|  | RASR | 5` CCAGGCTTTCGGCAAATTCC 3` | 20 | 1496 – 1515 | Ristaino et al. 2001 |
|  | RAS1F | 5’ CCATCGGTGTTGACTTTGTG 3` | 20 | 970 – 989 | This report |
|  | RAS1R | 5` GGCGTACAATTTGGAGCTTG 3` | 20 | 1113 – 1132 | This report |
|  | RAS2F | 5` CCCGCGTGATTTCCTATTTA 3` | 20 | 1151 – 1170 | This report |
|  | RAS2R | 5` AGCGGTCCATCACTTAGCTC 3` | 20 | 1378 – 1397 | This report |
|  |  |  |  |  |  |
| *PiAVR2* | AVR2F1 | 5` GCCACTCTGTGGTGTGGTTA 3` | 20 | 2 – 21 | Gilroy *et al*. 2011 |
|  | AVR2R2 | 5` CGCCGAGCTCTTAACTCCT 3` | 19 | 503 – 521 | Gilroy *et al*. 2011 |
|  | AVR2F4 | 5` ATGCGTCTCGCCTACATTTT 3` | 20 | 161 – 180 | Gilroy *et al*. 2011 |
|  | qRT-PCR-R | 5` CTTTTCCGTGACCTCTTTAGC 3` | 21 | 425 – 445 | Gilroy *et al*. 2011 |
|  |  |  |  |  |  |
| P3 | F3 | 5` ATGGTAGAGCGTGGGAATCAT 3` | 21 | 2893 – 2913 | Griffith and Shaw 1998 |
|  | R3 | 5` AATACCGCCTTTGGGTCCATT 3` | 21 | 4178 – 4198 | Griffith and Shaw 1998 |
|  | P3H4F | 5` TTGGTGATACAATTTTAATTTCTGC 3` | 25 | 3265 – 3289 | This report |
|  | P3H6R | 5` TTGTGATTATAGGTTTTTGATTCG 3` | 24 | 3733 – 3756 | This report |
|  |  |  |  |  |  |
| HERB-1 | nad11F | 5` AAAGTTAATCCTAAATGTTTTATAGGGTAAGTG 3` | 33 | 28057 – 28089 | This report |
|  | nad11R | 5` TACTTTAAAATCTATTGAAGAATCTGATCTTTG 3` | 33 | 28204 – 28236 | This report |

^a^Location of primer in original DNA sequence, GenBank Accession U304704 for *IRRas*, original publication (Gilroy et al. 2011) for *PiAVR2*, GenBank Accession U17009 for P3 mitochondrial region, and GenBank Accession AY894835 for HERB-1 mitochondrial region.

^b^IRF1 was utilized in samples for which IRF/IRR did not produce an amplicon, as it produced a slightly shorter product.
