# Supplementary material for: Historic Late Blight Outbreaks Caused by a Widespread Dominant Lineage of Phytophthora infestans (Mont.) de Bary
Source: PLoS One. 2016 Dec 28;11(12):e0168381. doi: 10.1371/journal.pone.0168381 (PMC5193357; doi:10.1371/journal.pone.0168381)
Supplement: S3 Table — Summary statistics for all runs: within population statistics: mean number of alleles and mean genetic diversity; between sample statistics: mean number of alleles, mean genetic diversity, Fst, shared allele distance, (δμ) 2, and maximum likelihood coefficient of admixture. (DOCX) [file pone.0168381.s008.docx]

**S3 Table. Prior distributions for Do It Yourself Approximate Baysian Computation (DIYABC) scenarios.** Summary statistics for all runs: within population statistics: mean number of alleles and mean genetic diversity; between sample statistics: mean number of alleles, mean genetic diversity, Fst, shared allele distance, (δµ) ^2^, and maximum likelihood coefficient of admixture.

| Parameter | Shape | Min ― Max |
| --- | --- | --- |
| Population size |  |  |
| US Famine Era | Uniform | 10000 ― 200000 |
| South America | Uniform | 10000 ― 300000 |
| Mexico | Uniform | 10000 ― 1000000 |
| Unsampled population | Uniform | 10000 ― 2000000 |
|  |  |  |
| Time since divergence^a^ |  |  |
| t1: Two populations | Uniform | 10000 ― 200000 |
| t2: Two or three populations | Uniform | 10000 ― 500000 |
| t3: Inclusion of unsampled populations | Uniform | 10000 ― 10000000 |
|  |  |  |
| Admixture events |  |  |
| Admixture rate | Uniform | 0.001 ― 0.999 |
| ta1: Timing since admixture event | Uniform | 1000 ― 300000 |
| ta2: Timing since admixture event | Uniform | 1000 ― 1000000 |
|  |  |  |
| Nucleotide sequence evolution^b^ |  |  |
| Mean/individual mutation rate | Uniform | 1.00x10^-10^/1.00 x10^-11^ ― 1.00x10^-8^/1.00x10^-7^ |

^a^ In order to guide the construction of trees, priors related to the time since divergence and admixture events were defined, such that t2>t1, t3>t2, t3>ta2, t3>ta1, ta2>t2, t2>ta1, and t3>t1

^b^ The stepwise mutation model was used as the basis of the substitution model
