# Supplementary material for: Historic Late Blight Outbreaks Caused by a Widespread Dominant Lineage of Phytophthora infestans (Mont.) de Bary
Source: PLoS One. 2016 Dec 28;11(12):e0168381. doi: 10.1371/journal.pone.0168381 (PMC5193357; doi:10.1371/journal.pone.0168381)
Supplement: S4 Table — (DOCX) [file pone.0168381.s009.docx]

**S4 Table. Diversity statistics for clone corrected microsatellite data for all 12 loci within populations of *Phytophthora infestans.***

| **Population**^a^ | ***n***^b^ | **MLG**^c^ | **eMLG(SE)** ^d^ | **H**^e^ | **G**^f^ | **Hexp**^g^ | **Evenness** | **Ia**^h^ | $\bar{\boldsymbol{r}}$***_d_***^i^ |
| --- | --- | --- | --- | --- | --- | --- | --- | --- | --- |
| USHist | 33 | 30 | 10(na) | 3.40 | 30 | 1 | 1 | 0.617 | 0.0730 |
| EUHist | 13 | 13 | 10(7.3e-8) | 2.56 | 13 | 1 | 1 | 1.207 | 0.1240 |
| US-1 | 19 | 19 | 10(2.51e-7) | 2.94 | 19 | 1 | 1 | 0.995 | 0.0933 |
| SA | 30 | 29 | 10(0.13e-6) | 3.37 | 29 | 1 | 1 | 2.29 | 0.2170 |
| CA | 23 | 23 | 10(5.03e-7) | 3.14 | 23 | 1 | 1 | 3.469 | 0.3269 |
| MEX | 26 | 26 | 10(1.09e-6) | 3.26 | 26 | 1 | 1 | 0.226 | 0.0214 |
| USAGG | 30 | 30 | 10(na) | 3.40 | 30 | 1 | 1 | 0.987 | 0.0927 |
| IRE | 9 | 9 | 9(0) | 2.20 | 9 | 1 | 1 | 1.039 | 0.0987 |

^a^ Populations sampled included USHist: US historic herbarium samples (1855-1958); EUHist: European historic herbarium samples (1846-1970); US-1 lineage (1931-1995); SA: South American (1913-2009); CA: Central American (1941-2003); MEX: Mexican (1948-1998), USAGG: US Aggressive lineages (1992-2014); IRE: Ireland (1993-1999).

^b^*n*: number of individuals (not clone corrected);

^c^MLG: number of multilocus genotypes (MLG);

^d^eMLG: expected number of MLGs at smallest size of at least ten; SE: Standard error;

^e^H: Shannon-Weiner Index of MLG diversity;

^f^G: Stoddart and Taylor Index of MLG diversity;

^g^Hexp: Nei’s 1978 expected heterozygosity;

^h^Ia: Index of Association;

^i^$\bar{r}$*_d_*: standardized index of association
