# Supplementary material for: Historic Late Blight Outbreaks Caused by a Widespread Dominant Lineage of Phytophthora infestans (Mont.) de Bary
Source: PLoS One. 2016 Dec 28;11(12):e0168381. doi: 10.1371/journal.pone.0168381 (PMC5193357; doi:10.1371/journal.pone.0168381)
Supplement: S5 Table — (DOCX) [file pone.0168381.s010.docx]

**S5 Table. Population statistics, diversity indices, and neutrality tests among *Phytophthora infestans* populations using *ras, PiAVR2* and the P3 mitochondrial region.**

|  | Sample summaries | | | | | Parameter estimates | | Test of neutrality | |
| --- | --- | --- | --- | --- | --- | --- | --- | --- | --- |
| Locus/Population | *L* | *n* | *s* | *h* | *k* | π(SE) | θw(SE) | Tajima’s D statistic | Fu’s Fs statistic |
| *ras*^1^ |  |  |  |  |  |  |  |  |  |
| US Historic | 680 | 24 | 2 | 3 | 0.60 | 0.06(0.06) | 0.54(0.40) | 0.27(ns) | 0.28(ns) |
| EU Historic | 680 | 4 | 1 | 2 | 0.50 | 0.05(0.06) | 0.55(0.55) | -0.61(ns) | 0.17(ns) |
| US-1/Ib | 680 | 28 | 1 | 2 | 0.48 | 0.05(0.05) | 0.26(0.26) | 1.36(ns) | 1.50(ns) |
| South America | 680 | 40 | 8 | 3 | 2.48 | 0.25(0.15) | 1.88(0.83) | 0.91(ns) | 5.57(ns) |
| Central America | 680 | 18 | 1 | 2 | 0.21 | 0.02(0.03) | 0.29(0.29) | -0.53(ns) | -0.01(ns) |
| Mexico | 680 | 26 | 2 | 3 | 0.68 | 0.07(0.06) | 0.52(0.39) | 0.59(ns) | 0.55(ns) |
| US Aggressive Lineages | 680 | 40 | 8 | 4 | 3.51 | 0.35(0.20) | 1.88(0.83) | 2.47(ns) | 5.81(ns) |
| Ireland | 680 | 14 | 8 | 5 | 2.09 | 0.21(0.14) | 2.52(1.24) | -0.64(ns) | 0.18(ns) |
| Pooled | 680 | 194 | 10 | 8 | 1.90 | 0.19(0.12) | 1.71(0.64) | 0.26 (ns) | 1.11(ns) |
|  |  |  |  |  |  |  |  |  |  |
| *PiAVR2* |  |  |  |  |  |  |  |  |  |
| US Historic | 200 | 36 | 1 | 2 | 0.44 | 2x10^-3(^2x10^-3^) | 0.24(0.24) | 1.19(ns) | 1.48(ns) |
| EU Historic | 200 | 2 | 1 | 2 | 0.53 | 3x10^-3^(3x10^-3^) | 0.35(0.35) | 1.30(ns) | 1.03(ns) |
| US-1/Ib | 200 | 18 | 1 | 2 | 0.37 | 2x10^-3^(2x10^-3^) | 0.29(0.29) | 0.48(ns) | 0.79(ns) |
| South America | 200 | 28 | 1 | 2 | 0.35 | 2x10^-3(^2x10^-3^) | 0.26(0.26) | 0.57(ns) | 0.95(ns) |
| Central America | 200 | 10 | 1 | 2 | 0.36 | 2x10^-3(^2x10^-3^) | 0.35(0.35) | 0.02(ns) | 0.42(ns) |
| Mexico | 200 | 4 | 1 | 2 | 0.67 | 3x10^-3^(4x10^-3^) | 0.55(0.55) | 1.63(ns) | 0.54(ns) |
| US Aggressive Lineages | 200 | 34 | 1 | 2 | 0.47 | 2x10^-3(^2x10^-3^) | 0.25(0.25) | 1.39(ns) | 1.59(ns) |
| Ireland | 200 | 6 | 0 | 1 | 0.00 | 0.00(0.00) | 0.00(0.00) | ND | ND |
| Pooled | 200 | 146 | 1 | 2 | 0.41 | 2x10^-3(^2X10^-3^) | 0.18(0.18) | 1.35(ns) | 2.02(ns) |
|  |  |  |  |  |  |  |  |  |  |
| P3 |  |  |  |  |  |  |  |  |  |
| US Historic | 379 | 11 | 0 | 1 | 0.00 | 0.00(0.00) | 0.00(0.00) | ND | ND |
| EU Historic | 379 | 8 | 0 | 1 | 0.00 | 0.00(0.00) | 0.00(0.00) | ND | ND |
| US-1/Ib | 379 | 7 | 0 | 1 | 0.00 | 0.00(0.00) | 0.00(0.00) | ND | ND |
| South America | 379 | 23 | 3 | 3 | 1.29 | 3x10^-3^(2x10^-3^) | 0.81(0.52) | 1.49(ns) | 2.03(ns) |
| Central America | 379 | 15 | 0 | 1 | 0.00 | 0.00(0.00) | 0.00(0.00) | ND | ND |
| Mexico | 379 | 13 | 1 | 2 | 0.15 | 4x10^-4(^6x10^-4^) | 0.32(0.32) | -1.15(ns) | -0.54(ns) |
| US Aggressive Lineages | 379 | 8 | 3 | 2 | 1.61 | 4x10-3(3x10-3) | 1.16(0.78) | 1.60(ns) | 2.99(ns) |
| Ireland | 379 | 7 | 3 | 2 | 1.43 | 4x10^-3^(3x10^-3^) | 1.22(0.84) | 0.76(ns) | 2.51(ns) |
| Pooled | 379 | 92 | 4 | 4 | 1.20 | 3x10-3(2x10-3) | 0.79(0.43) | 1.07(ns) | 2.01(ns) |

*l*, consensus sequence length; *n*, sample size (number of isolates for mitochondrial locus and number of alleles for nuclear locus); *s*, segregating nucleotide sites; *h*, haplotypes; *k*, average number of pairwise nucleotide differences; π, average number of base differences per site; SE, standard error; θw, population mean mutation rate of Watterson’s θ estimator. ND, not determined because there was no polymorphism; ns, not significant.

^1^: For the purposes of acquiring sample summaries, parameter estimates, and neutrality tests, the largest nonrecombining partition was utilized for all values except *l*.
