# Supplementary material for: Historic Late Blight Outbreaks Caused by a Widespread Dominant Lineage of Phytophthora infestans (Mont.) de Bary
Source: PLoS One. 2016 Dec 28;11(12):e0168381. doi: 10.1371/journal.pone.0168381 (PMC5193357; doi:10.1371/journal.pone.0168381)
Supplement: S6 Table — (DOCX) [file pone.0168381.s011.docx]

**S6 Table. Distribution of haplotypes and base substitution events in nuclear and mitochondrial genes of *Phytophthora infestans*.**

| Locus |  | |  |  |  | Nuclear (*Intron Ras* + *ras*)^a^ | | | | | | |  |  |  |  | Nuclear (*PiAVR2*) | | |  |  | Mitochondrial (P3) | | | |
| --- | --- | --- | --- | --- | --- | --- | --- | --- | --- | --- | --- | --- | --- | --- | --- | --- | --- | --- | --- | --- | --- | --- | --- | --- | --- |
| Position in  GenBank Database Accession^b^ |  |  |  |  |  |  | * |  |  |  |  |  |  |  |  |  |  |  |  |  |  |  |  |  |  |
|  |  |  |  |  |  |  |  | 1 | 1 | 1 | 1 | 1 |  |  |  |  |  |  |  |  |  | 3 | 3 | 3 | 3 |
|  | 5 | 5 | 6 | 6 | 6 | 7 | 7 | 0 | 0 | 2 | 3 | 3 |  |  |  |  | 2 |  |  |  |  | 4 | 4 | 5 | 5 |
|  | 6 | 9 | 0 | 1 | 6 | 0 | 1 | 2 | 6 | 1 | 0 | 5 |  |  |  |  | 5 |  |  |  |  | 0 | 7 | 1 | 6 |
|  | 1 | 6 | 3 | 0 | 0 | 1 | 6 | 8 | 4 | 3 | 3 | 1 |  |  |  |  | 3 |  |  |  |  | 4 | 6 | 9 | 8 |
|  |  |  |  |  |  |  |  |  |  |  |  |  |  |  |  |  |  |  |  |  |  |  |  |  |  |
| Position in  combined  consensus |  |  |  |  |  |  |  |  |  |  |  |  |  |  |  |  |  |  |  |  |  |  |  |  |  |
|  |  |  | 1 | 1 | 1 | 2 | 2 | 3 | 3 | 4 | 5 | 6 |  |  |  |  |  |  |  |  |  | 1 | 1 | 2 | 2 |
|  | 6 | 9 | 0 | 1 | 6 | 0 | 1 | 1 | 4 | 9 | 8 | 3 |  |  |  |  |  |  |  |  |  | 1 | 8 | 3 | 8 |
|  | 3 | 8 | 5 | 2 | 2 | 3 | 8 | 2 | 8 | 7 | 7 | 5 |  |  |  |  | 8 |  |  |  |  | 7 | 9 | 2 | 1 |
|  |  |  |  |  |  |  |  |  |  |  |  |  |  |  |  |  |  |  |  |  |  |  |  |  |  |
| Site Number |  |  |  |  |  |  |  |  |  | 1 | 1 | 1 |  |  |  |  |  |  |  |  |  |  |  |  |  |
|  | 1 | 2 | 3 | 4 | 5 | 6 | 7 | 8 | 9 | 0 | 1 | 2 |  |  |  |  | 1 |  |  |  |  | 1 | 2 | 3 | 4 |
|  |  |  |  |  |  |  |  |  |  |  |  |  |  |  |  |  |  |  |  |  |  |  |  |  |  |
| Site Type^c^ | t | v | v | t | t | t | t | v | t | t | t | v |  |  |  |  | v |  |  |  |  | v | v | v | v |
| Character Type | i | i | i | i | - | i | - | i | i | i | i | i |  |  |  |  | - |  |  |  |  | i | - | i | i |
| Substitution  Type | x | x | x | x | x | x | x | r | s | r | r | r |  |  |  |  | r |  |  |  |  | s | s | r | r |
| Consensus | C | G | A | A | A | G | G | G | G | G | T | T |  |  |  |  | T |  |  |  |  | T | A | G | A |
|  |  |  |  |  |  |  |  |  |  |  |  |  |  |  |  |  |  |  |  |  |  |  |  |  |  |
| Haplotype (Count) |  |  |  |  |  |  |  |  |  |  |  |  |  |  |  |  |  |  |  |  |  |  |  |  |  |
| rasH1 (109) | **.** | **.** | **.** | **.** | **.** | **.** | **.** | **.** | **.** | **.** | **.** | **.** |  |  | avrH1 (105) |  | A |  |  | mH1 (66) |  | **.** | **.** | T | **.** |
| rasH2 ( 20) | T | T | C | G | **.** | A | **.** | **.** | **.** | A | C | A |  |  | avrH2 ( 41) |  | **.** |  |  | mH2 (24) |  | A | **.** | **.** | C |
| rasH3 ( 1) | T | **.** | C | G | **.** | A | **.** | **.** | **.** | A | C | A |  |  |  |  |  |  |  | mH3 ( 1) |  | **.** | C | T | **.** |
| rasH4 ( 1) | T | T | C | G | **.** | A | **.** | **.** | **.** | A | C | **.** |  |  |  |  |  |  |  | mH4 ( 1) |  | **.** | **.** | **.** | **.** |
| rasH5 ( 2) | **.** | **.** | **.** | **.** | G | **.** | **.** | **.** | A | **.** | **.** | **.** |  |  |  |  |  |  |  |  |  |  |  |  |  |
| rasH6 ( 1) | **.** | **.** | **.** | **.** | **.** | **.** | A | **.** | A | **.** | **.** | **.** |  |  |  |  |  |  |  |  |  |  |  |  |  |
| rasH7 ( 5) | **.** | **.** | **.** | **.** | **.** | **.** | **.** | T | A | **.** | **.** | **.** |  |  |  |  |  |  |  |  |  |  |  |  |  |
| rasH8 ( 1) | **.** | **.** | **.** | **.** | **.** | **.** | **.** | T | **.** | **.** | **.** | **.** |  |  |  |  |  |  |  |  |  |  |  |  |  |
| rasH9 ( 51) | **.** | **.** | **.** | **.** | **.** | **.** | **.** | **.** | A | **.** | **.** | **.** |  |  |  |  |  |  |  |  |  |  |  |  |  |
| rasH10 ( 2) | **.** | **.** | **.** | **.** | **.** | **.** | **.** | **.** | **.** | A | C | A |  |  |  |  |  |  |  |  |  |  |  |  |  |
| rasH11 ( 1) | **.** | **.** | **.** | **.** | **.** | **.** | **.** | **.** | **.** | **.** | C | **.** |  |  |  |  |  |  |  |  |  |  |  |  |  |

^a^ Haplotype distribution does not include the presence of two observed heterozygous sites in the *ras* region, at positions 1178 (G/T) and 1187 (T/C). Historic samples sequenced poorly around this region, and as such the region was cut from subsequent analyses.

^b^Numbering in vertical columns indicates the site number of GenBank accession no. U30474 for *ras* (Chen and Roxby 1996), U17009 for mitochondrial loci (Paquin et al. 1997; Avila-Adame et al. 2007), and published sequence from Gilroy et al (2011) for *PiAVR2*. Sites 561-716 are located in intron 1, sites 1064 -1351 are located in introns and exons 3-6, respectively of the *ras* gene. Sites 3404-3568 are located in the P3 region (*rpl 14, rpl5* genes).

^c^t, transitions; v, transversions; i, phylogenetically informative sites; -, uninformative sites; r, nonsynonymous (i.e., replacement) substitutions; s, synonymous subsititutions; x, non-coding region.

*Indicated *ras* locus was not previously observed as a SNP by Gómez-Alpizar et al. (2007).
