# Supplementary material for: Historic Late Blight Outbreaks Caused by a Widespread Dominant Lineage of Phytophthora infestans (Mont.) de Bary
Source: PLoS One. 2016 Dec 28;11(12):e0168381. doi: 10.1371/journal.pone.0168381 (PMC5193357; doi:10.1371/journal.pone.0168381)
Supplement: S7 Table — (DOCX) [file pone.0168381.s012.docx]

**S7 Table. Population subdivision of *Phytophthora infestans* populations at *ras* locus according to Hudson’s test statistics Ks (upper right matrix) and Kst (lower left matrix).**

|  | **USHist** | **EUHist** | **US-1** | **SA** | **CA** | **MEX** | **USAGG** | **IRE** |
| --- | --- | --- | --- | --- | --- | --- | --- | --- |
| **USHist** |  | 0.593ns | 0.534ns | 1.980* | 0.436* | 0.636ns | 2.903** | 1.219** |
| **EUHist** | -0.005ns |  | 0.478ns | 2.664ns | 0.241* | 0.655ns | 4.049ns | 2.087ns |
| **US-1** | -0.007ns | 0.040ns |  | 1.843* | 0.374ns | 0.568ns | 2.709*** | 1.068* |
| **SA** | 0.044* | -0.011ns | 0.046* |  | 2.017ns | 1.961* | 3.507ns | 2.676ns |
| **CA** | 0.101* | 0.344* | 0.050ns | 0.031ns |  | 0.484** | 3.043** | 1.127ns |
| **MEX** | -0.013ns | -0.017ns | 0.005ns | 0.053* | 0.117** |  | 2.855*** | 1.229** |
| **USAGG** | 0.130** | 0.011ns | 0.138*** | 0.024ns | 0.099** | 0.139*** |  | 3.784ns |
| **IRE** | 0.084** | -0.001ns | 0.078* | -0.015ns | 0.052ns | 0.093** | 0.012ns |  |

Significance was evaluated by performing 1000 permutations including incompatible sites and recombinant regions.

Significance values: ns, not significant; *, 0.01 < P < 0.05; **, 0.001 < P < 0.01; ***, P < 0.001
