# Supplementary material for: Historic Late Blight Outbreaks Caused by a Widespread Dominant Lineage of Phytophthora infestans (Mont.) de Bary
Source: PLoS One. 2016 Dec 28;11(12):e0168381. doi: 10.1371/journal.pone.0168381 (PMC5193357; doi:10.1371/journal.pone.0168381)
Supplement: S8 Table — (DOCX) [file pone.0168381.s013.docx]

**S8 Table**. **Locus, haplotype, isolate identities and population sampled for modern and historic isolates of *Phytophthora infestans*.**

| Locus | Haplotype (Frequency) | Isolates^a^ | Populations^b^ |
| --- | --- | --- | --- |
| *ras* | H1 (109) | US0186656a, US0186674a, US0186680a, US0186868a, US0186890a, US0186891a, US0186905a, FH206a, FH206b, H288a, US0186882a, US0186882b, 796293a, K79a, 94-1a, 95-6a, 95-6b, US920141a, 188.1.1a, US0186927a, US0186927b, FH287a, US0186807a, US0186807b, US0187006a, US0187006b, CUP4a, PA222a, K125a, K126a, PER802a, PER832a, FH292a, FH292b, B189b, B193b, B217b, B219b, BOL3b, PCZ007b, PCZ050b, PCZ026a, PCZ033a, PCZ098a, PCZ118a, PHU006a, POX004a, PCO038a, PPA008a, PPU003a, EC3092a, EC3154a, EC3154b, EC3300a, DR-4a, DR-4b, ZBa, ZBb, ZEa, ZEb, 52a, 52b, 61a, 61b, 141a, 141b, 151a, 151b, 152a, 152b, PIC97180a, PIC97224a, PIC97370a, PIC97605a, PIC97620a, PIC97620b, PIC97630a, PIC97652a, PIC97652b, PIC98301a, PIC98366a, PIC98372a, P445a, 94-52b, 920159b, 94-53a, 2.1.3a, 93-3a, 94-11-2a, 94-8-4b, 94-19b, 97-24b, 98-97b, 268.1.5b, TN-070-Ab, TNFL-2b, NY09-nightshade-Ab, PSUTomAb, BL2009P4b, ND884-1b, 21A/93a, 21A/93b, 12/94a, 18/94a, 18/94b, 6/95a, 6/95b, 31/95a, 31/95b | USHIST, EUHIST, US-1, SA, CA, MEX, USAGG, IRE |
|  | H2 (20) | B189a, B193a, B217a, B219a, BOL3a, PCZ007a, PCZ050a, 94-52a, 920159a, 94-8-4a, 94-19a, 97-24a, 98-97a, 980059a, 342.1.1a, NY09-nightshade-Aa, PSUTomAa, BL2009P4a, ND884-1a, 3/99a | SA, USAGG, IRE |
|  | H3 (1) | 16/99a | IRE |
|  | H4 (1) | 268.1.5a | USAGG |
|  | H5 (2)  H6 (1)  H7 (5)  H8 (1)  H9 (51)  H10 (2)  H11 (1) | PIC97605b, PIC98301b  US0186905b  94-53b, 2.1.3b, 93-3b, 94-11-2b, 342.1.1b  980059b  US0186656b, US0186674b, US0186680b, US0186868b, US0186872a, US0186872b, US0186890b, US0186891b, FH288b, 796293b, DBN11a, DBN11b, K79b, 94-1b, US92014b, 188.1.1b, H287b, CUP4b, PA222b, K125b, K126b, PER802b, PER832b, PCZ026b, PCZ033b, PCZ098b, PCZ118b, PHU006b, POX004b, PCO038b, PPA008b, PPU003b, EC3092b, EC3300b, US0186832a, US0186832b, PIC97180b, PIC97207a, PIC97207b, PIC97224b, PIC97370b, PIC97388a, PIC97388b, PIC97630b, PIC98366b, PIC98372b, P445b, 94-22a, 94-22b, 12/94b, 3/99b  TN-070-Aa, TNFL-2a  16/99b | MEX  USHIST  USAGG  USAGG  USHIST, EUHIST, US-1, SA, CA, USAGG, IRE  USAGG  IRE |
|  |  |  |  |

| *PiAVR2* | H1 (105) | US0186680a, US0186680b, US0186905a, US0186905b, US0186656a, 796293a, 796293b, FH206a, FH206b, US0186674a, US0186674b, US0186668a, US0186668b, US0186964a, US0186964b, US0186897a, US0186891a, US0186882a, US0186890a, US0186987a, US0186967a, US0186967b, FH283a, US0186979a, US0186868a, FH219a, K22a, DBN11a, K43a, K42b, UPS9a, US0186816a, US0186816b, US0187022a, US0186832a, US0187006a, US0187006b, US0186927a, US0186927b, FH287a, CUP4a, CUP4b, PA222a, P445a, K125a, K125b, 94-1a, US920141a, US920141b, 188.1.1a, 920159a, 94-55a, 94-52a, 94-22a, 94-11-2a, 93-3a, 93-3b, 94-53a, 94-53b, 2.1.3a, 342.1.1a, 342.1.1b, 268.1.5a, 268.1.5b, 980059a, 980059b, TN-070-Aa, TNFL-2a, NY09-nightshade-Aa, BL2009P4a, PSUTomAa, ND884-1a, PIC97630a, US0186968a, FH292a, B189a, B193a, B217a, B219a, EC3094a, EC3094b, PCZ026a, PCZ026b, PCZ033a, PCZ033b, PCZ098a, PCZ098b, PCZ118a, PCZ118b, PHU006a, PHU006b, PCZ050a, PCZ050b, PPA008a, PPA008b, 12/94a, 12/94b, 21A/93a, 21A/93b, 31/95a, 31/95b, 52a, 52b, 141a, 141b | CA, EUHIST, IRE, MEX, SA, USAGG, USHIST, US-1 |
| --- | --- | --- | --- |
|  | H2 (41) | US0186656b, US0186897b, US0186891b, US0186882b, US0186890b, US0186987b, FH283b, US0186979b, US0186868b, US0186928a, US0186928b, FH219b, K22b, DBN11b, UPS9b, US0187022b, US0186832b, FH287b, PA222b, P445b, 94-1b, 188.1.1b, 920159b, 94-55b, 94-52b, 94-22b, 94-11-2b, 2.1.3b, TN-070-Ab, TNFL-2b, NY09-nightshade-Ab, BL2009P4b, PSUTomAb, ND884-1b, PIC97630b, US0186968b, H292b, B189b, B193b, B217b, B219b | CA, EUHIST, MEX, SA, USAGG, USHIST, US-1 |

|  |  |  |  |
| --- | --- | --- | --- |
| *P3* | H1 (66) | FH288, US0186905, US0186656, 796293, US0186674, US0186891, US0186890, US0186987, FH283, US0186979, US0186868, K22, UPS1, FH222, DBN11, UPS2, K79, UPS9, DBN2, K126, US0186927, K125, 94-1, PER802, US0186968, FH292, PCZ007, PCZ050, PER810, PCA014, PPL018, PCA001, US0187022, US0186832, ZE, 52, 141, 152, 42, 92, 201, 211, 71, 221, 81, 91, 151, PIC97180, PIC97630, PIC97652, PIC97224, PIC98301, PIC98305, PIC98366, PIC97388, PIC97605, PIC98372, PIC97620, PIC97370, 94-53, RS2009P1, TNFL-2, TN-070-A, BL2009P4, 12/94, 16/99 | CA, EUHIST, IRE, MEX, SA, USAGG, USHIST, US-1 |
|  | H2 (24) | BOL9, B217, B219, B189, B193, EC3094, EC3199, EC3253, EC3298, EC3300, EC3154, PCZ026, PPU003, PCZ098, PCZ118, PPA008, 94-52, 342-1-1, 980059, 3/99, 21A/93, 15/99, 6/95, 18/94 | IRE, SA, USAGG |
|  | H3 (1) | PIC97207 | MEX |
|  | H4 (1) | PHU006 | SA |

^a^ Each isolate is suffixed with a lowercase letter (a or b) indicating phase.

^b^ Populations sampled included USHist: US historic herbarium samples (1855-1958); EUHist: European historic herbarium samples (1846-1970); US-1 lineage (1931-1995); SA: South American (1913-2009); CA: Central American (1941-2003); MEX: Mexican (1948-1998), USAGG: US Aggressive lineages (1992-2014); IRE: Ireland (1993-1999).
