# Supplementary material for: Historic Late Blight Outbreaks Caused by a Widespread Dominant Lineage of Phytophthora infestans (Mont.) de Bary
Source: PLoS One. 2016 Dec 28;11(12):e0168381. doi: 10.1371/journal.pone.0168381 (PMC5193357; doi:10.1371/journal.pone.0168381)
Supplement: S9 Table — (DOCX) [file pone.0168381.s014.docx]

**S9 Table. Isolation model (IM) parameter estimates for *Phytophthora infestans* populations from US Historic (USHist), EU Historic (EUHist), Mexican (MEX), US-1 and South America (SA) based on variation in the *ras* or *PiAVR2* nuclear loci.**

| **Parameter** | **USHist (1)**  **vs**  **EUHist (2)** | **USHist (1) vs**  **MEX (2)** | **USHist (1) vs**  **SA (2)** | **US-1 (1)**  **vs**  **MEX(2)** | **US-1 (1)**  **vs**  **SA (2)** |
| --- | --- | --- | --- | --- | --- |
| *IRRas* |  |  |  |  |  |
| θ_1_ | 0.64 (0.10, 6.85) | 1.18 (0.15, 7.49) | 0.83 (0.15, 6.22) | 0.36 (0.04, 3.43) | 0.46 (0.06, 2.29) |
| θ_2_ | 2.86 (0.09, 10.48) | 0.83 (0.13, 6.89) | 1.05 (0.18, 11.03) | 1.32 (0.16, 7.20) | 1.20 (0.24, 8.8212.47) |
| θ_A_ | 5.03 (0.20, 10.61) | 3.47 (0.15, 7.80) | 9.82 (0.67, 20.56) | 3.68 (0.17, 7.65) | 9.91 (0.71, 20.56) |
| T | 5.01 (0.08, 9.76) | 4.71 (0.04, 9.74) | 4.70 (0.28, 9.74) | 5.00 (0.08, 9.75) | 4.40 (0.23, 9.73) |
| m_1_ | 4.59 (0.16, 9.77) | 6.34 (0.28, 9.86) | 4.62 (0.18, 9.74) | 5.03 (0.22, 9.78) | 4.85 (0.23, 9.74) |
| m_2_ | 6.63 (0.50, 9.86) | 5.70 (0.23, 9.83) | 6.25 (0.81, 9.82) | 7.02 (0.57, 9.88) | 6.18 (0.74, 9.83) |
|  |  |  |  |  |  |
| *PiAVR2* |  |  |  |  |  |
| θ_1_ | 0.35 (0.04, 3.35) | 0.29 (0.04, 1.96) | 0.41(0.05, 3.62) | 0.38 (0.04, 4.33) | 0.46 (0.05, 4.28) |
| θ_2_ | 1.40 (0.08, 6.70) | 3.88 (0.14, 10.54) | 0.51 (0.05, 4.39) | 3.3 (0.11, 10.5) | 0.73 (0.07, 5.29) |
| θ_A_ | 3.23 (0.14, 6.87) | 5.24 (0.22, 10.62) | 2.37 (0.11, 5.00) | 5.19 (0.21, 10.62) | 2.60 (0.11, 5.65) |
| T | 4.87 (0.02, 9.75) | 5.06 (0.20, 9.75) | 4.88 (0.02, 9.75) | 5.05 (0.12, 9.76) | 4.75 (0.02, 9.74) |
| m_1_ | 5.05 (0.22, 9.77) | 4.04 (0.15, 9.68) | 5.87 (0.34, 9.83) | 4.45 (0.17, 9.74) | 5.78 (0.30, 9.83) |
| m_2_ | 6.99 (0.62, 9.88) | 7.17 (1.06, 9.88) | 6.49 (0.51, 9.86) | 6.87 (0.66, 9.87) | 6.54 (0.45, 9.86) |
|  |  |  |  |  |  |

For the purposes of parameter estimation, the inheritance scalar was set to 1 for *ras* and *PiAVR2*. In each population pairing, the first population listed is considered to be population 1, and the second to be population 2. The parameters are as follows: θ_1_: Mean population mutation rate (theta) for population 1; θ_2_: Mean population mutation rate (theta) for population 2; θ_A_: Mean population mutation rate (theta) for the ancestral population; t: Mean estimation of divergence time between the populations from a common ancestor; m_1_: Mean number of migrants into population 1; m_2_: Mean number of migrants into population 2. Values in parentheses indicate the estimated point at which 2.5% of the area of the prior distribution lies to the left or right, respectively. IM runs were set up using a 500,000 step burn-in and a recording period of 10 million steps. To achieve convergence, runs utilized metropolis coupling with 30 chains and a geometric increment model, with geometrical increment parameters of 0.8 and -0.9. A total of three runs using different random seeds was used for each population pairing to ensure convergence. The average of the parameters with the most optimal run for each pairing is displayed. Initial maxima for each parameter was set to 10 with the exception of θ_2_ and θ_A_, which were determined by the program.
