# Supplementary material for: Historic Late Blight Outbreaks Caused by a Widespread Dominant Lineage of Phytophthora infestans (Mont.) de Bary
Source: PLoS One. 2016 Dec 28;11(12):e0168381. doi: 10.1371/journal.pone.0168381 (PMC5193357; doi:10.1371/journal.pone.0168381)
Supplement: S10 Table — Probabilities listed are the highest values as calculated over a 10-sample logistic regression. (DOCX) [file pone.0168381.s015.docx]

**S10 Table.** **Posterior probabilities and confidence intervals from three *Phytophthora infe*stans populations.** Probabilities listed are the highest values as calculated over a 10-sample logistic regression.

| **Populations^a^** | **Scenario** | **Probability^b^** | **95% CI** |
| --- | --- | --- | --- |
| USHist-SA-MEX | 1 | 0.0077 | [0.0000, 0.0312] |
|  | **2** | **0.5040** | **[0.4471, 0.5608]** |
|  | 3 | 0.0569 | [0.0117, 0.1020] |
|  | 4 | 0.0130 | [0.0000, 0.0574] |
|  | 5 | 0.4926 | [0.4684, 0.5168] |
|  | 6 | 0.0008 | [0.0000, 0.0244] |
|  | 7 | 0.0017 | [0.0000, 0.0252] |
| US1-SA-MEX | 1 | 0.1753 | [0.1669, 0.1837] |
|  | 2 | 0.1343 | [0.1104, 0.1581] |
|  | **3** | **0.3463** | **[0.3306, 0.3621]** |
|  | 4 | 0.3072 | [0.2749, 0.3394] |
|  | 5 | 0.0428 | [0.0220, 0.0636] |
|  | 6 | 0.0012 | [0.0000, 0.0075] |
|  | 7 | 0.0145 | [0.0083, 0.0207] |
| USHist-EUHist-SA | 1 | 0.2008 | [0.1691, 0.2325] |
|  | 2 | 0.0000 | [0.0000, 0.0996] |
|  | 3 | 0.0000 | [0.0000, 0.0996] |
|  | 4 | 0.0035 | [0.0000, 0.0704] |
|  | 5 | 0.0000 | [0.0000, 0.0519] |
|  | 6 | 0.3046 | [0.2802, 0.3290] |
|  | 7 | 0.0000 | [0.0000, 0.0393] |
|  | 8 | 0.0000 | [0.0000, 0.0441] |
|  | **9** | **0.5107** | **[0.4439, 0.5775]** |
|  | 10 | 0.0251 | [0.0000, 0.0687] |
|  | 11 | 0.0363 | [0.0089, 0.0636] |

^a^ Populations included USHist: US historic herbarium samples (1855-1958); EUHist: European historic herbarium samples (1846-1970); US-1 lineage (1931-1995); SA: South American (1913-2009); MEX: Mexican (1948-1998); USAGG: US Aggressive lineages (1992-2014).

^b^Probabilities listed are the highest values as calculated over a 10-sample logistic regression.
